# Unimodal to multimodal: a systematic review of predictive machine learning models for valvular heart diseases

**DOI:** 10.3389/fcvm.2026.1855775

**Published:** 2026-07-01

**Authors:** Valentine Ojonugwa Idakwo, Caren Strote, Christian Goelz, Qasrina Shafei, Thomas J. Stocker, Jörg Hausleiter, Solveig Vieluf

**Affiliations:** 1 Department of Medicine I, LMU University Hospital, LMU Munich, Munich, Germany; 2 DZHK (German Centre for Cardiovascular Research), Partner Site Munich Heart Alliance, Munich, Germany; 3 relAI – Konrad Zuse School of Excellence in Reliable AI, Munich, Germany; 4 Faculty of European Campus Rottal-Inn, Deggendorf Institute of Technology (DIT), Pfarrkirchen, Germany

**Keywords:** machine learning, multimodal machine learning, predictive machine learning, procedural outcomes, risk stratification, valvular heart disease

## Abstract

**Objectives:**

We aimed to synthesize existing evidence on predictive machine learning (ML) models for valvular heart disease (VHD) and examine how these models have been applied across clinical tasks, data modalities and validation settings.

**Background:**

ML is gaining traction for improving cardiovascular care, particularly in the management of VHDs. However, empirical evidence on how ML models handle the multimodal complexity of valvular pathologies remain sparse.

**Methods:**

We conducted a systematic review according to the Preferred Reporting Items for Systematic Reviews and Meta-Analyses (PRISMA) guidelines searching PubMed, Web of Science, and Embase from 2014 to 2025. We included articles that developed ML for clinical prediction in VHD patients. (PROSPERO: CRD42025644167).

**Results:**

We identified 195 studies that met the inclusion criteria. Seventy-five studies (38.5%) developed single-lesion models for aortic stenosis. Retrospective datasets were used in 86% of the included studies and 79% relied on internal validation. Sixteen studies (8.2%) developed multimodal models, integrating different types of ML input data. The multimodal models demonstrated a 6.3 percentage point increase in average performance across tasks compared to their unimodal counterparts within the same cohort.

**Conclusion:**

Across the literature, unimodal ML models for VHDs demonstrate promising performance for disease detection, patient stratification, and risk prediction, but multimodal approaches are emerging with potential advantages for procedural planning and outcome forecasting. Translation to clinical practice will require large, multicenter datasets to validate and standardize data-driven VHD management.

**Systematic Review Registration:**

https://www.crd.york.ac.uk/PROSPERO/view/CRD42025644167.

## 1 Introduction

Valvular heart disease (VHD) is a primary contributor to cardiovascular morbidity and mortality, affecting approximately 2.5% of the general population and over 13% of adults older than 75 years globally; this burden is expected to increase as societies age (1–3). Untreated VHD often leads to increased heart failure hospitalizations, decreased quality of life and premature mortality. Evidence suggests that intervening at earlier stages of significant VHD leads to better long-term outcomes (4). Additionally, clinical prediction in VHD is challenging due to the diversity of valve types (aortic, mitral, tricuspid, and pulmonic), lesion characteristics (stenosis vs. regurgitation), patient heterogeneity, and the existence of multi-valve disease, which may involve distinct diagnostic approaches, symptom profiles, and therapeutic targets.

Machine learning (ML), a subfield of artificial intelligence (AI) capable of learning complex non-linear patterns and generating predictions from high-dimensional datasets, enhances diagnostic precision, optimizes patient selection and provides a data-driven framework to guide treatment decision. Crucially, ML offers the unique capacity to synthesize the disparate multimodal data sources that are fundamental to the multidisciplinary Heart Team's decision-making process. For this study, a predictive ML model is defined as a data-driven model that uses clinical data to estimate health outcomes, adopting the domain classification system previously established for clinical prediction (5). These include diagnosis, prognosis, disease progression, readmission risks, risk assessment, complication risks, treatment response, and mortality prediction. Current ML models for VHDs have started to show promising performance, with some reporting high area-under-the-curve (AUC) values (often ≥ 0.80) for predicting relevant endpoints (6). Despite these promising developments, translation of the developed models into clinical practice is sparse (6). The rapid growth of research in this area has also led to a fragmented body of literature, highlighting the need for a systematic synthesis to integrate and summarize existing evidence. This systematic review aims to synthesize how predictive ML models for VHD are being developed, validated, and reported, in patients with VHD, comparing unimodal and multimodal approaches where reported, with primary outcomes of model performance, validation methodology, and data modality use, across original model development studies. We also aim to characterize the primary domains of predictive ML application and identify critical gaps in the current evidence base. Key term definitions are detailed in Supplementary Table S5.

## 2 Materials and methods

### 2.1 Protocol and registration

This review adheres to the Preferred Reporting Items for Systematic Reviews and Meta-analyses (PRISMA) (7) guidelines. We registered our search strategy and protocol in advance on PROSPERO (CRD42025644167).

### 2.2 Data source and search strategy

Three databases: PubMed, Web of Science, and Embase were searched on November 1, 2025. We conducted a title and abstract search of each of the databases for articles published between January 1, 2014, and November 1, 2025, using a combination of MeSH terms and multiple synonym-search. The complete search strategy is provided in Supplementary Table S6.

We imported our search results into a platform for managing systematic reviews; Rayyan (8) and removed all duplicate entries. Two authors (VI and CS) independently screened the title and abstract based on the defined inclusion and exclusion criteria. Disagreements were resolved by consensus or arbitration by another author (CG). The two authors (VI and CS) subsequently reviewed the full texts and resolved discrepancies via consensus and or arbitration by a third author (CG). A summary of the eligibility criteria is provided in Supplementary Methods.

### 2.3 Data extraction and analysis

We developed a structured data extraction form to record the key characteristics of the included studies. These included: publication year, type of VHD, primary predictive task, primary data modality, study setting, study design, validation source, reproducibility, and explainability. In multimodal models, we also extracted the type of data fusion employed. Due to the variability in study populations, primary outcomes, ML algorithms, and data sources, we did not conduct a meta-analysis.

### 2.4 Risk of bias assessment

We used the Prediction model Risk Of Bias Assessment Tool (PROBAST) + AI (9) for assessing the risk of bias (RoB) of the included studies. Two authors (VI and QS) independently assessed the RoB of the included studies for accuracy and consistency (Supplementary Figure S4, Supplementary Table S2).

### 2.5 Ethical considerations

As this study is a systematic review of previously published literature and does not involve the primary collection of individual patient data or direct interaction with human subjects, institutional review board (IRB) approval and informed consent were not required. All included studies were conducted in accordance with the Declaration of Helsinki and had obtained their own respective ethical approvals.

## 3 Results

### 3.1 Search results

**Figure 1:**
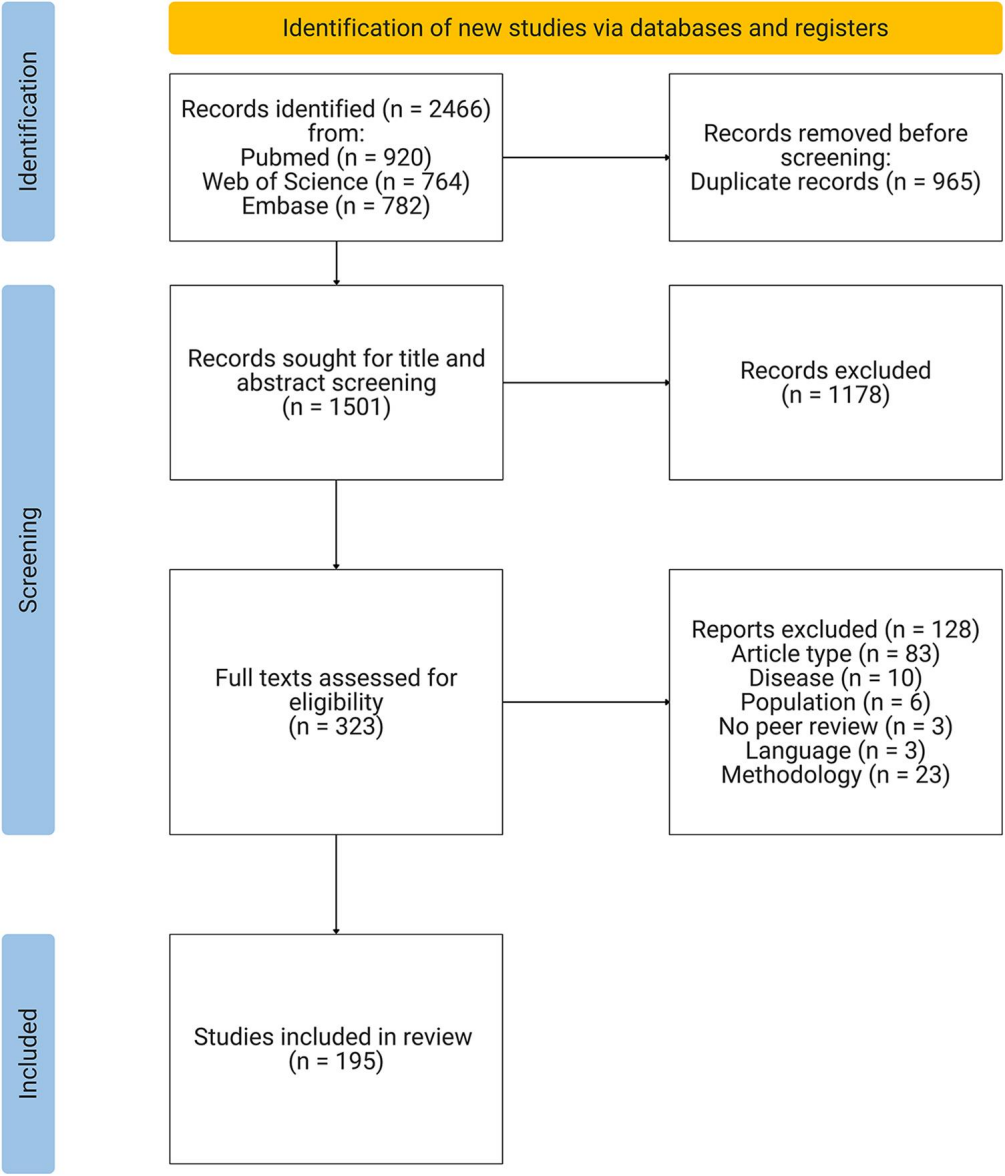
PRISMA flow diagram of the study selection process.

A total of 2466 articles were identified in the initial search, of which 965 duplicates were removed. Following an abstract and full text screening, 195 articles were included in this review (10–202). Figure 1 shows the PRISMA flow of the review process. The publication frequency of included studies increased across the period analyzed. Specifically, 90% (*n* = 176) of the studies were published between 2021 and 2025, compared to 10% (*n* = 19) published between 2015 and 2020. The total count and annual distribution of included studies are contained in Supplementary Figure S1, and the corresponding data is contained in Supplementary Table S1.

### 3.2 Study setting and design

Model development relied on data from a single clinical center in 50.8% of studies (*n* = 99), with 23.1% (*n* = 45) utilizing data generated from multiple centers. The remaining 26.2% (*n* = 51) used datasets obtained from multiple secondary sources like medical websites and training databases. Most studies (86.2% *n* = 168) used retrospective datasets, whereas 11.8% (*n* = 23) used prospective datasets and 1.5% (*n* = 3) used a combination of retrospective and prospective datasets, where the model was developed on a retrospective dataset and validated on a prospective one. The study design was not specified in one of the studies (48). The sample sizes used for model development varied across the included studies, with a mean of 16,855 (SD = 72,665). A total of 64.1% (*n* = 125) of studies used a sample size of 1,000 patients or fewer. Through 2020, the mean sample size was 3,300 (SD = 7,920); from 2021 onward, the mean was 18,329 (SD = 76,343) (see Supplementary Figure S3). CNN-based models were the most frequently used models in the included studies. Detailed information regarding model characteristics is provided in the Supplementary Results.

### 3.3 Valvular heart disease type and predictive task

**Figure 2:**
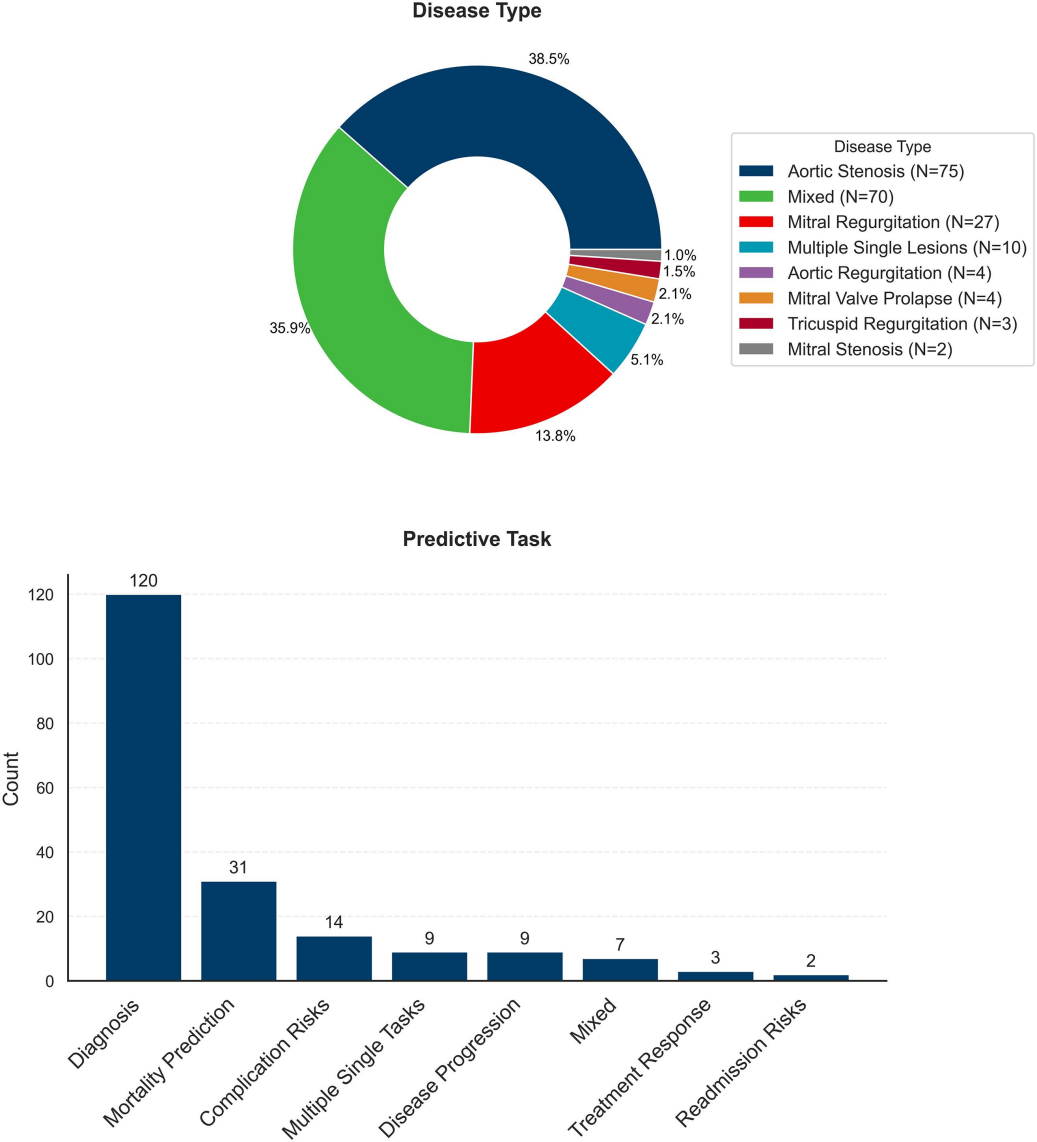
Distribution of studies based on VHD type and predictive task. “Mixed” denotes models trained to address multiple diseases or predictive tasks using a single unified model; “Multiple” refers to separate, single-lesion or task-specific models each addressing distinct VHD or predictive task respectively.

**Figure 3:**
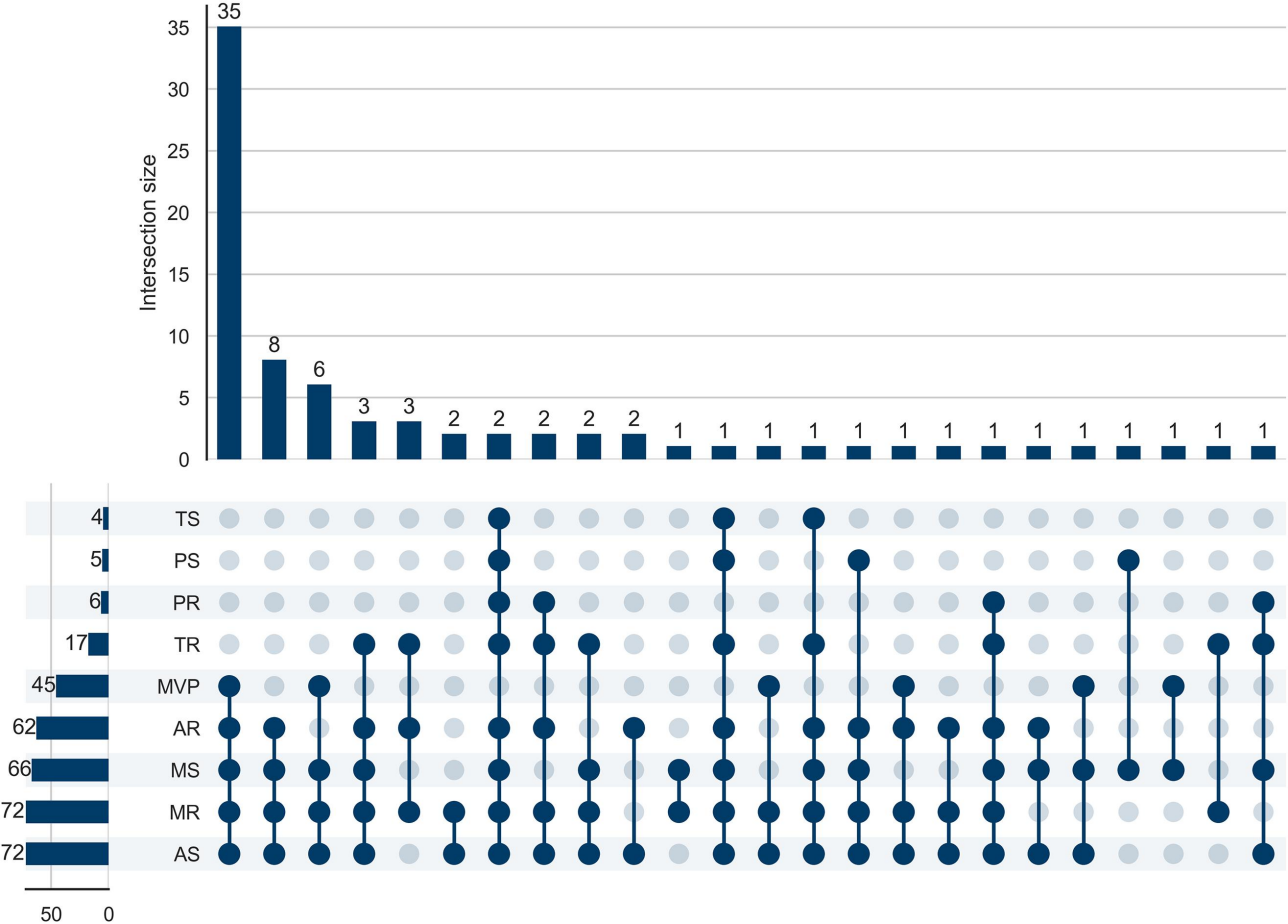
Disease combinations for multiple-disease models. AR, aortic regurgitation; AS, aortic stenosis, MR, mitral regurgitation; MS, mitral stenosis; MVP, mitral valve prolapse; PR, pulmonary regurgitation; PS, pulmonary stenosis; TR, tricuspid regurgitation; TS, tricuspid stenosis.

Seventy-five studies (38.5%) developed single-lesion models for aortic stenosis. In this study, we classify studies as mixed when a single model is applied to multiple VHDs or prediction tasks, and as multiple when separate models are developed for different diseases or tasks. Using this convention, 70 studies (35.9%) developed mixed models in which a single model addressed multiple diseases, whereas 10 studies (5.1%) developed multiple models each targeting a different VHD. For instance, Castela Forte et al. (25) developed separate models using 88 peri-operative variables to predict five-year mortality in patients who underwent aortic and mitral valve surgeries. Figure 2 shows the distribution of VHD types and predictive tasks, while Figure 3 illustrates the disease combinations for all mixed and multiple models.

For the primary predictive task, most of the included studies (61.5%, *n* = 120) were for diagnosis. Overall, 16 studies addressed more than one predictive task, either using mixed models (*n* = 7) or multiple models (*n* = 9). For instance, Erdogan et al. (41) developed a model to predict major adverse cardiovascular events (MACE), including myocardial infarction, cerebrovascular events and mortality, in AS patients within 30 days of transcatheter aortic valve implantation (TAVI). Gomes et al. (46) developed separate models to predict five distinct outcomes in AS patients after TAVI: mortality, stroke, major vascular complications, paravalvular leakage and the need for new pacemaker implantation.

The included studies disproportionately targeted left-sided VHD. This imbalance was more pronounced for tasks vital to interventional decision-making, such as the prediction of complication risks, treatment response, and readmission risk, where every model (100%) focused on left-sided VHDs. In contrast, right-sided VHD models were uncommon across predictive tasks and were most frequently represented in mortality prediction, where they accounted for 6.4% of models.

### 3.4 Data modalities

**Figure 4:**
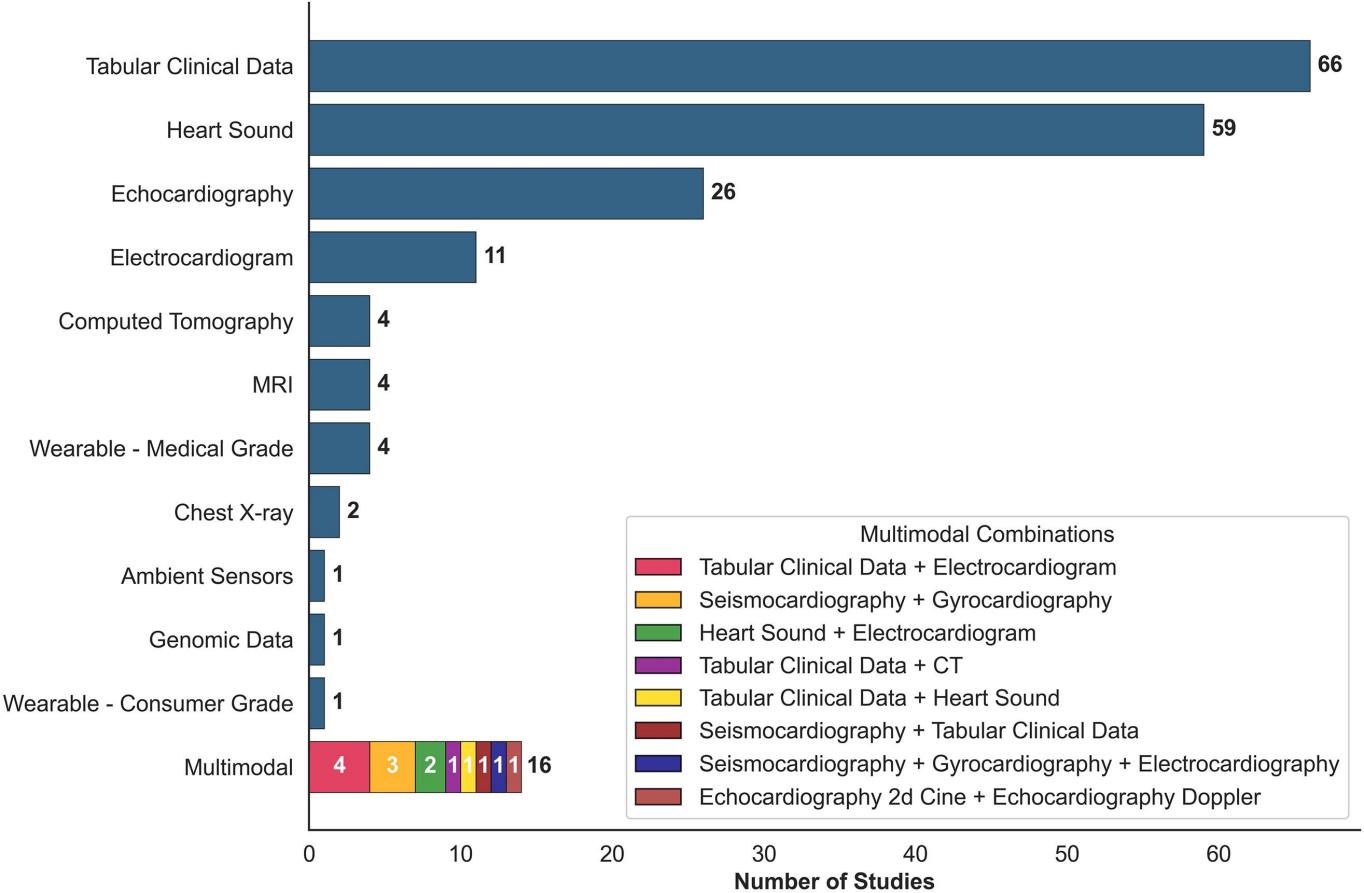
Data source distribution of included studies. CT, computed tomography; MRI, magnetic resonance imaging, x-ray, radiograph.

Unimodal models were reported in 179 studies (91.8%), and multimodal models in 16 (8.2%) (Figure 4 and Supplementary Table S3). All the studies that used multimodal models were published in 2020 or later. The data sources for the models included: tabular clinical data, echocardiography, chest radiographs, cardiac magnetic resonance (CMR), electrocardiogram (ECG), computed tomography (CT), ambient sensors, genomic data, wearables, and heart sounds. Modality use differed by predictive task. Diagnosis was mostly performed using heart sound recordings (49.2%), followed by echocardiography (20.0%) with smaller contributions from ECG (7.5%) and other modalities (Supplementary Table S3). In contrast, tabular clinical data predominated in all non-diagnostic tasks, including mortality prediction (93.6%), complication risk prediction (78.6%), disease progression (55.6%), and all models for readmission risk (100%) and treatment response (100%). Cross-sectional imaging was infrequently used and was confined to a minority of models for complication risk (CT 7.1%; MRI 7.1%) and mortality prediction (CT 3.2%).

Additionally, we identified a subset of studies that we classified as unimodal with multisource learning, in which handcrafted variables were manually extracted from heterogeneous sources (e.g., electronic health records, laboratory results, clinical history, imaging reports) and combined into a single structured input. Because no model operated directly on the raw data streams and fusion occurred only after manual feature engineering, these studies were not considered true multimodal models for the purposes of this review. For example, Hausleiter et al. (50) extracted 18 handcrafted pre-procedural features from clinical, laboratory, echocardiographic, and medication history data and fed them into an extreme gradient boosting (XGBoost) model to predict mortality in MR patients.

Brüggemann et al. (24) compared unimodal tabular models, unimodal models with multisource learning combining tabular data with handcrafted pre-procedural CT variables, and a multimodal model fusing raw CT with tabular inputs for mortality prediction in AS patients. The handcrafted CT variables comprised 15 radiologist-derived transcatheter aortic valve replacement (TAVR) planning measures, including aortic valve calcification burden, annular and left ventricular outflow tract (LVOT) geometry, and ascending aorta anatomy and calcification. Performance was comparable between the multimodal model and the best unimodal model with multisource learning (AUROC 0.725 vs. 0.723), whereas the best unimodal tabular model achieved an AUROC of 0.689.

In the papers that developed multimodal models, 5 used early data fusion, 7 used intermediate data fusion, and 4 used late data fusion. Of the 16 multimodal studies reviewed, 12 developed models for a single disease (10 for AS, 1 for MR, and 1 for AR). The remaining 4 studies developed either mixed or multiple models; specifically, 2 studies developed separate models for AS and MR, while 1 study developed separate models for AS, MS, AR and MR and 1 study developed a model for AS, MS, TR and PR (See Supplementary Tables 2, 3).

**Figure 5:**
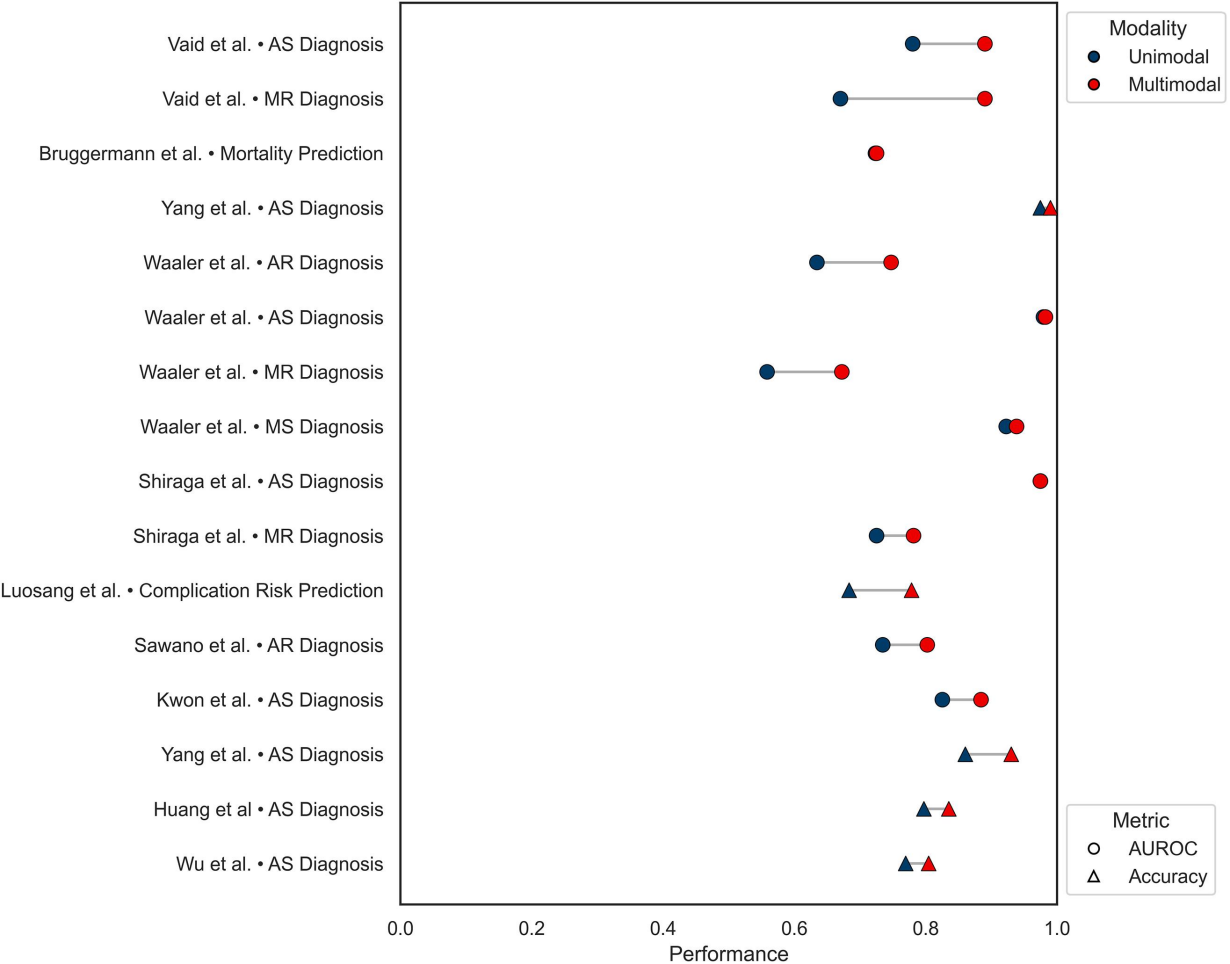
Comparative performance of multimodal and unimodal models in representative studies. AR, aortic regurgitation; AS, aortic stenosis; MR, mitral regurgitation; MS, mitral stenosis.

In 11 studies, the performance of the developed multimodal models was compared to that of the similar unimodal models using the same patient cohorts, reporting an average improvement of 6.3% across performance metrics (AUROC in 6 studies, accuracy in 5). The performance gains were greater when structured data (tabular clinical variables such as demographics, comorbidities, labs, and medications) were integrated with unstructured sources (raw signals or images such as heart sounds, ECG waveforms, or imaging) (8.0%) than when only unstructured modalities were combined (4.0%). The largest incremental gains were observed for MR diagnosis when tabular clinical data were combined with ECG (+22%) (132) or heart sound recordings (+11%) (137) relative to unimodal comparators within the same cohorts. In contrast, AS diagnosis showed no improvement when heart sounds were combined with ECG compared with ECG alone (+0%) (113). For mortality prediction, combining tabular clinical data with CT yielded a minimal gain (+0.2%) (24). Figure 5 shows the comparative performance of multimodal and unimodal models, demonstrating that the multimodal models achieved equal or higher performance. Given substantial heterogeneity in clinical tasks, performance metrics, populations, and model architectures, results should be interpreted as a descriptive summary. Further comparison of the multimodal and unimodal models is shown in Supplementary Table S3.

### 3.5 Risk of bias (RoB)

Nearly half of the included studies exhibited a high or unclear risk of bias regarding participant selection in both model development (51.3%) and evaluation (48.2%). The high RoB for participant selection was largely driven by non-representative sampling from public or challenge datasets, often curated from medical websites rather than consecutive clinical cohorts, the use of single center data for model development and evaluation, and by restrictive eligibility criteria. The summary RoB and the individual RoB are contained in Supplementary Figure S4 and Supplementary Table S2 respectively.

## 4 Discussion

This systematic review assessed predictive ML models for VHDs across 195 studies. These studies used several inputs spanning clinical variables, imaging, physiological signals and heart sound data to predict outcomes in VHD patients. Over the 11-year period, the number and scale of predictive ML studies for VHDs increased, indicating expanding interest in the clinical utility of these tools. The principal findings are: (1) model development remains concentrated in left-sided VHDs, with no right-sided models identified for complication risk, treatment response, or readmission prediction and only limited right-sided representation in diagnosis, mortality prediction, and disease progression; (2) the evidence is dominated by diagnostic ML applications; (3) despite the availability of heterogeneous inputs, the field remains predominantly unimodal, with comparatively limited adoption of multimodal modeling despite improvement across reported metrics of the multimodal models; and (4) multiple studies used “unimodal models with multisource learning,” in which handcrafted variables were manually extracted from heterogeneous sources and manually engineered into a single tabular input, yet direct comparisons between these handcrafted multisource approaches and models that fuse raw data streams remain limited.

### 4.1 Disease focus

Our review reveals a dominant left-sided anatomical and clinical skew in the current ML landscape for VHDs. AS was the primary focus addressed in nearly three-quarters of the included studies, followed by a secondary concentration on MR. Few studies developed models for the right-sided pulmonic and tricuspid valve diseases. While this distribution aligns with historical mortality patterns, where aortic and mitral pathologies account for the vast majority of VHD-related deaths (1), it exposes a significant evidence gap on the right sided heart and the evolving need of the structural Heart Team. The heavy emphasis on AS likely reflects not only its high clinical burden but also the therapeutic maturity of the TAVR evidence base and the high volume of data available for model training in these patients (203, 204). This emphasis also spans across predictive tasks, with ML used on echocardiography to diagnose AS (19), and a separate ML model was used to predict 1-year mortality from clinical data in AS patients (77). Importantly, the widespread adoption of TAVR, the relatively standardized thresholds for intervention, and the availability of large longitudinal registries such as the STS-ACC TVT registry (205) and the Heart Valve Society Aortic Valve Database (206) have created a particularly data-rich environment for model development and validation at scale. In contrast, other valvular lesions often involve more heterogenous disease presentations, treatment pathways, and data sources, which can make large-scale model development and validation more challenging despite the growing body of research in these areas (207). As registry infrastructure and prospective data collection continue to expand across the full spectrum VHDs, similar opportunities for ML development are likely to emerge. From a mechanistic perspective, predictive ML has favored AS because it offers a particularly well-defined prediction problem characterized by a common disease, a frequently performed intervention, measurable procedural outcomes, and repeated follow-up (208). This combination makes AS exceptionally well suited for retrospective modeling and external validation. Hence, the predominance of left-sided VHD models within the current ML literature likely reflects a more direct translational pathway rather than an intrinsic performance advantage. Consequently, the current distribution of predictive ML studies across VHDs may be viewed not only as a reflection of disease prevalence, but as an indicator of therapeutic maturity and data availability.

Given the rarity of pulmonic valve diseases, and TS in adult patients, these lesions are infrequently encountered in routine echocardiography cohorts and thus rarely modelled in ML studies (209, 210). In contrast, TR is prevalent, affecting an estimated 75% of adults with mild TR and 4% of individuals over 75 with moderate or severe TR globally. Historically, the relative neglect of TR in ML literature is perhaps not surprising as the tricuspid valve has been known as the “forgotten valve”, with research and innovation lagging behind the left-sided heart valves (211). Our review confirms that the tricuspid valve remains the “forgotten valve” of the digital era. This gap is especially critical given the rapid expansion of transcatheter tricuspid valve interventions (TTVI). More broadly, this pattern reflects a wider disparity across regurgitant valvular lesions, as predictive modelling for mitral, tricuspid, and aortic regurgitation remains less mature, owing to the greater heterogeneity in disease mechanisms, more variable timing of intervention, and less standardized outcome frameworks (212). These factors can make the development, validation, and comparison of ML models more challenging across studies.

Our findings also highlight a significant limitation of the current literature: the tendency to model heart valves in isolation. In interventional cardiology, left-sided lesions often drive right-sided failure, and the success of a mitral intervention is frequently linked to the state of the right-sided heart valves (213). We argue that the current one-valve-one-model approach is physiologically incomplete. Future models must embrace a coupled modelling approach that utilizes the hemodynamic interplay across all four valves for clinical prediction.

### 4.2 Predictive task

On the primary predictive task, majority of the studies were primarily developed for diagnosis, far outnumbering those focusing on mortality prediction and other tasks. This emphasis on diagnostic applications aligns with prior analysis showing that ML is most often applied to diagnosis in clinical settings (214, 215). Most of the diagnostic models used heart sounds and echocardiography, highlighting an emphasis on improving early detection and screening.

Further understanding of the clinical utility of these findings can be achieved by condensing the eight clinical task domains into a three-tier taxonomy based on the clinical patient pathway, in which Tier 1 focuses on diagnosis and detection; Tier 2 on disease progression (mortality, disease progression, prognosis, and baseline risk assessment); and Tier 3 on interventional prescription (treatment response, procedural complications, and readmission risks). The overrepresentation of diagnostic models suggests that researchers are targeting earlier pathway tasks rather than the nuanced clinical issues that are faced by the interventional team. Subsequently the sparse representation of domains vital for interventional planning such as treatment response, complication risks and readmission risks, underscores significant gaps in literature. In contemporary structural heart care, the central unmet need is often less the detection of VHD than estimation of the net clinical benefit of intervention for an individual patient. Collectively, our findings show a strong proof-of-principle that ML models can predict clinically relevant VHD outcomes across the structural heart pathway.

Only a handful of studies pursued multiple predictive tasks, highlighting the rarity of the multi-task approach. This suggests that ML predictive research in VHD patients has been largely siloed by task, which may be problematic given the multifaceted and longitudinal nature of outcomes in this population. For example, a siloed model that predicts mortality for AS without the risk of post-interventional complications might not fully address the needs of contemporary cardiovascular interventional practice. This siloed approach may also be insufficient as multivalve disease is becoming increasingly common due to population aging (216), a trend that necessitates a more comprehensive, multi-task approach to accurately capture the complexity of VHD.

### 4.3 Data modalities

A marked disparity exists between the inherent multimodality of VHDs and the unimodal nature of most current ML models. In contemporary structural heart practice, clinicians routinely integrate structured clinical information, imaging-derived anatomy and hemodynamics, and physiologic signals when determining diagnosis, timing of intervention, and procedural strategy. Valvular pathology simultaneously affects the heart's structure (annulus, leaflets), flow (doppler signatures, murmurs), electrophysiology, and general clinical presentation (217), such that models trained on a single modality typically only capture a limited representation of disease phenotype.

In contrast, multimodal architectures offer a principled approach to combine complementary signals and may improve data efficiency via shared representations (218). In our review, however, most models remained unimodal, and the use of multimodal input data were comparatively uncommon. Although unimodal models demonstrated robust utility, multimodal approaches showed incremental performance gains in the subset of studies that performed within-cohort head-to-head comparisons, consistent with prior studies (72, 132). The magnitude of improvement varied by predictive task and VHD, with the most pronounced gains observed in MR diagnosis when tabular clinical data were integrated with ECG, whereas other pairings showed little or no incremental benefit in specific settings. These findings suggest that incremental benefit from multimodality is not uniform and may depend on whether the added modality contributes nonredundant signal for the target outcome.

The inclusion of unimodal models with multisource learning in some studies reflects the perceived value of multimodal information but introduces potential limitations inherent to handcrafted feature engineering. Manual extraction can compress rich raw data into a small number of subjective measurements, potentially leading to substantial information losses vital for outcome prediction (219, 220). Additionally, human-in-the-loop measurements may introduce operator variability and center-specific practice patterns, complicating generalizability. Direct evidence comparing the advantages and trade-offs of truly multimodal models vs. unimodal models with multisource learning remains scarce.

The clinical relevance of multimodality depends on alignment to the interventional pathway. In this review, multimodal models were most frequently developed for diagnosis. In clinical settings, these models offer immediate impact for screening and triage; by incorporating a second data stream, they can better identify VHD and standardize point-of-care decision-making. By contrast, multimodal models for downstream outcomes most relevant to interventional prescription, such as procedural complications, treatment response, and readmission, remain limited. The potential of multimodal ML is well-supported by studies across various medical disciplines, which consistently demonstrate that fusing heterogeneous data yields more robust and accurate predictions (221–223). However, future research should prioritize multimodal models that use real-time available inputs and are validated through prospective assessments in structural heart clinical workflows.

### 4.4 Methodology

Beyond input modality, study design and data quality represent major determinants of clinical deployability. Across the included studies, the use of prospective datasets was uncommon, and most models were trained on retrospective, convenience cohorts. Such sampling may not reflect the broader VHD population encountered in contemporary practice and increases susceptibility to spectrum bias. In interventional cardiology, where device iterations, imaging protocols and procedural techniques evolve regularly, models trained on frozen retrospective samples are vulnerable to temporal dataset shift and at risk of becoming clinically obsolete. Reuse of the same limited cohorts across studies further compounds these concerns and has been associated with optimistic performance estimates and reduced generalizability when evaluated in independent settings (224, 225). The lack of external validation in most of the included studies also limits clinical deployability. Consistent with broader cardiovascular ML literature (226), the translational potential of predictive VHD models is constrained not only by architectural complexity but also by the limited use of external validation. The expansion of external validation across diverse populations and healthcare settings represents an important next step towards confirming model generalizability and facilitating the translation of these promising tools in routine clinical practice.

In this review, we observed that relatively few large, high-quality public datasets exist for VHD ML research, resulting in multiple studies often using similar data. This was largely common in studies that used heart sounds to diagnose VHDs, as the majority of them used the publicly available dataset by Yaseen et al. (227). This practice increases the likelihood that reported performance metrics reflect dataset-specific idiosyncrasies rather than disease-generalizable features. When such datasets are repeatedly used for development and benchmarking, apparent gains may reflect implicit adaptation to dataset-specific recording conditions, labeling conventions, or population mix, rather than true advances in clinical discrimination.

PROBAST + AI assessment additionally revealed methodological limitations across the included studies that moderate interpretation of reported performance metrics. The analysis domain carried the greatest burden of risk with most studies having high or unclear risk, reflecting widespread deficiencies in calibration reporting, inappropriate validation strategies, and insufficient sample sizes relative to model complexity. Participant-related risk was also considerable, with approximately half of studies rated high or unclear risk for both development and evaluation, driven predominantly by reliance on limited public datasets, single-center cohorts, and restrictive eligibility criteria that limit representativeness of real-world VHD populations. While Predictor and Outcome domains were comparatively well-handled, the systemic weaknesses in analysis and participant selection mean that reported performance estimates across all predictive tasks should be interpreted cautiously and are likely optimistic relative to what would be observed in prospective, independent clinical evaluation.

### 4.5 Clinical utility

In the near term, the most plausible benefit of predictive ML in VHD lies in the enhancement of diagnostic accuracy and the expansion of screening for VHDs. The sensitivity of standard auscultation by primary care physicians is low, leading to missed diagnoses and delayed referrals. In a study comparing detection rates in a real-world primary care setting, the AI-augmented system detected 92.3% of VHDs, whereas primary care providers detected 46.2% (228). By enabling mass screening in non-specialized settings, this capability ensures that patients with significant disease are flagged for echocardiography much earlier in the disease trajectory.

Once diagnosis has been established, the clinical challenge shifts to determining the optimal timing for intervention. Current practice relies on risk scores (e.g., EUROSCORE II) that were designed for surgical outcomes and often perform poorly on transcatheter therapies (229, 230). The ML studies for mortality prediction, baseline risk assessment, disease progression, and prognosis provide more accurate, patient-specific assessments. Furthermore, as the field moves beyond current achievements, the literature suggests the potential for substantial benefits from multimodal ML. Future models will utilize disparate data for holistic risk stratification, patient selection, and management.

Additionally, foundation models, including large language models integrated into large-scale multimodal AI systems are likely to become key enabling technologies as the field shifts towards holistic, personalized risk stratification (219, 231, 232). In VHD research and clinical decision support, foundation models could function as versatile computational backbones that can be adapted and fine-tuned for specific valvular lesions, rare phenotypes, or site-specific datasets, thereby reducing the need for *de novo* model development for each application. Concurrently, large-scale multimodal AI systems may facilitate the development of future “digital twin” frameworks, enabling individualized prognosis and procedural planning, and treatment optimization through the integration of heterogeneous clinical, imaging, and physiological data sources. However, their broader adoption will depend on overcoming major challenges, including high computational demands, limited transparency in model development, and the need for rigorous external validation before routine clinical use (231).

### 4.6 Study limitations

As a limitation, we were unable to conduct a meta-analysis across the included studies due to the heterogeneity of model architecture, clinical outcomes, data sources, and study designs. Hence there is no cumulative estimates for task-specific performance of the included studies. Furthermore, the reported performance gains are descriptive summaries rather than formal statistical comparisons, reflecting the diverse metrics and limited number of studies available for analysis. Additionally, while this review utilized a systematic methodology to identify predictive ML models for VHDs, the scope was restricted to PubMed, Web of Science and Embase databases. Consequently, relevant models published in alternative databases may not have been captured. The primary strengths of this review were the systematic and extensive search of 3 relevant databases, the application of a strict inclusion criteria in the evaluation of studies, and the use of reporting guidelines for systematic reviews.

## 5 Conclusion

This systematic review of 195 studies highlights the rapid growth of predictive ML in VHD, though research remains largely focused on diagnostic models for left-sided valve disease. Comparative evidence indicates that multimodal integration can improve performance in selected settings. However, true multimodal models remain underrepresented and are rarely evaluated against unimodal models with handcrafted multisource data. To enable clinical adoption in interventional practice, future work should prioritize holistic models aligned with the interventional pathway using decision-time inputs, robust external validation, and multicenter prospective evaluation.

## Data Availability

The original contributions presented in the study are included in the article/Supplementary Material, further inquiries can be directed to the corresponding author.
