# Supplementary material for: Unimodal to multimodal: a systematic review of predictive machine learning models for valvular heart diseases
Source: Front Cardiovasc Med. 2026 Jul 1;13:1855775. doi: 10.3389/fcvm.2026.1855775 (PMC13369510; doi:10.3389/fcvm.2026.1855775)
Supplement: Supplementary file 1 [file Datasheet1.docx]

Supplementary Document

**Unimodal to multimodal: a systematic review of predictive machine learning models for valvular heart diseases**

**
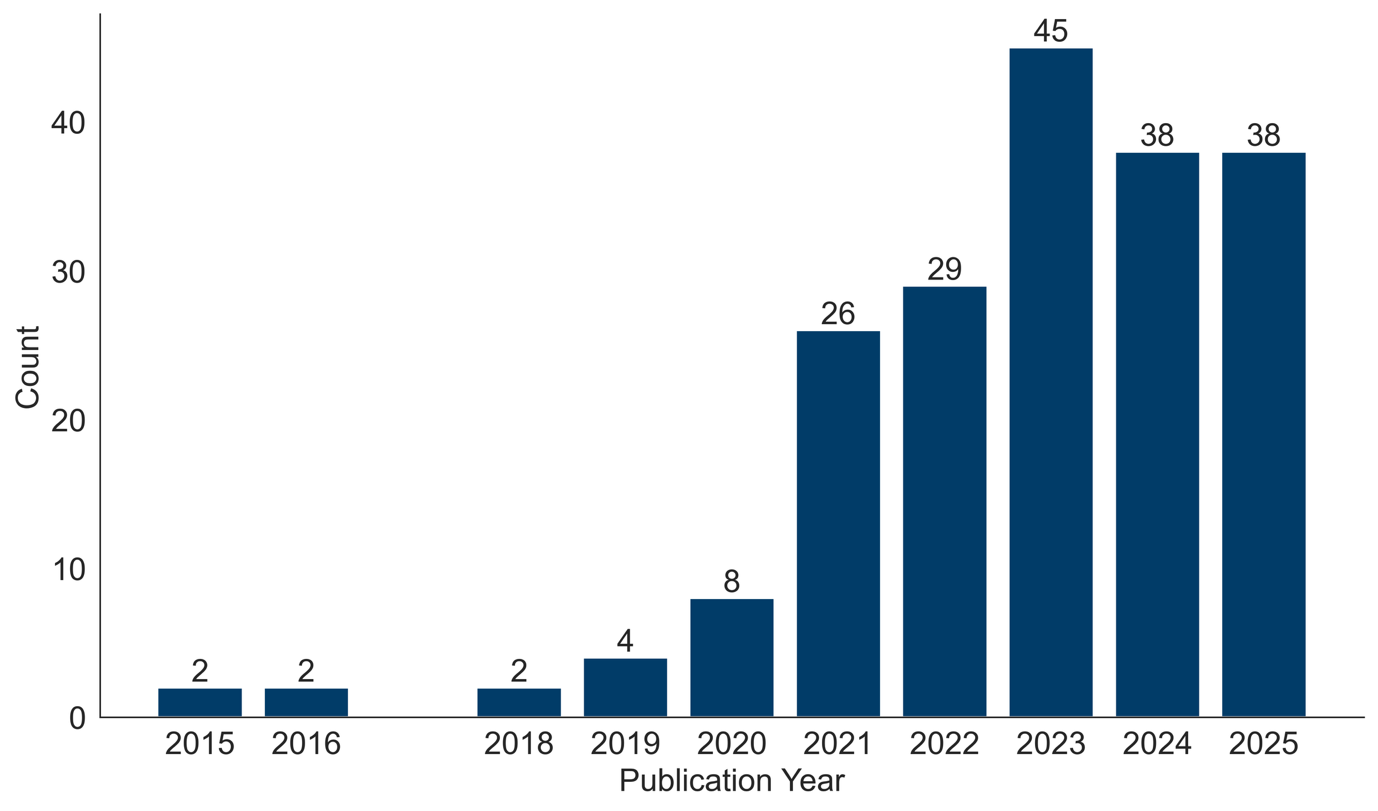
**

Supplementary Figure 1 | Publication year of included journals


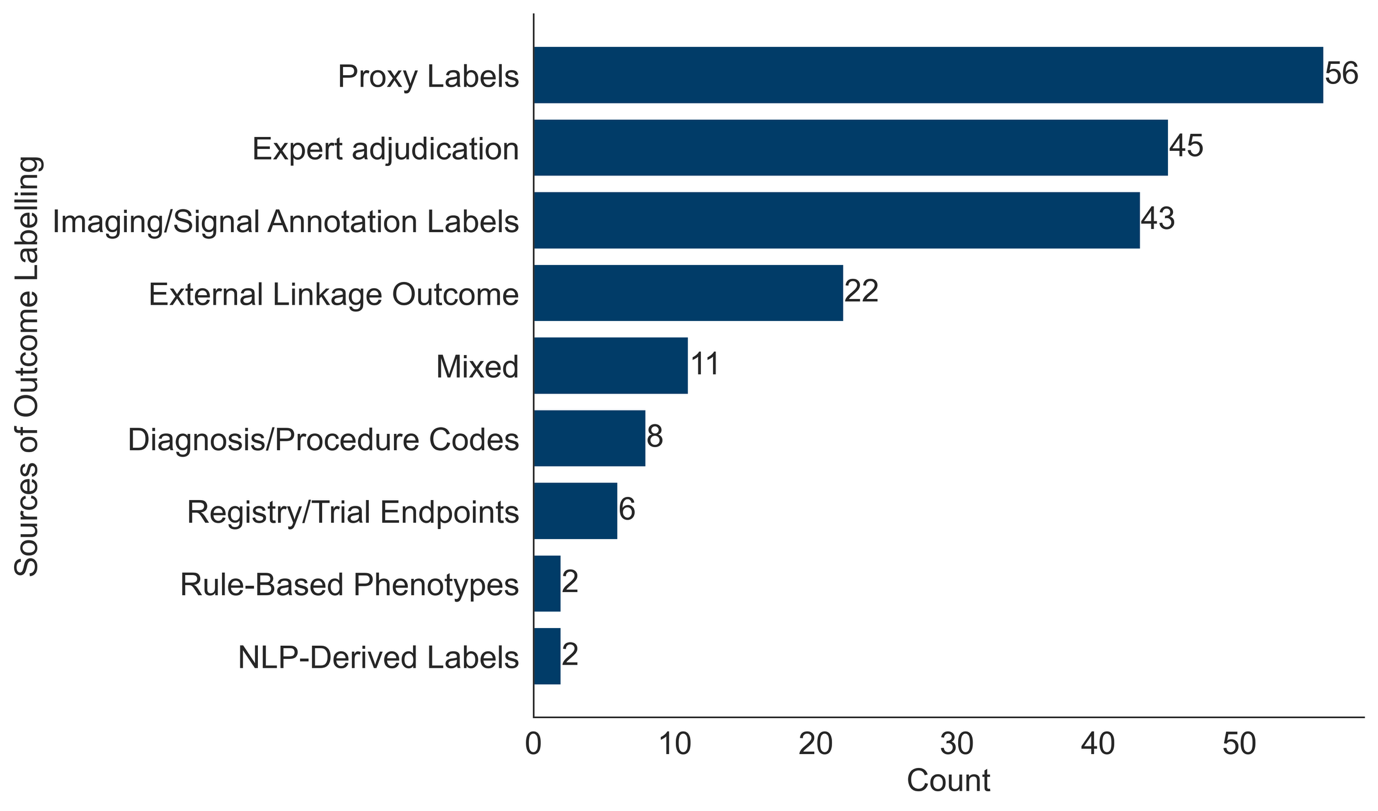


Supplementary Figure 2 | Sources of outcome labels of included studies.


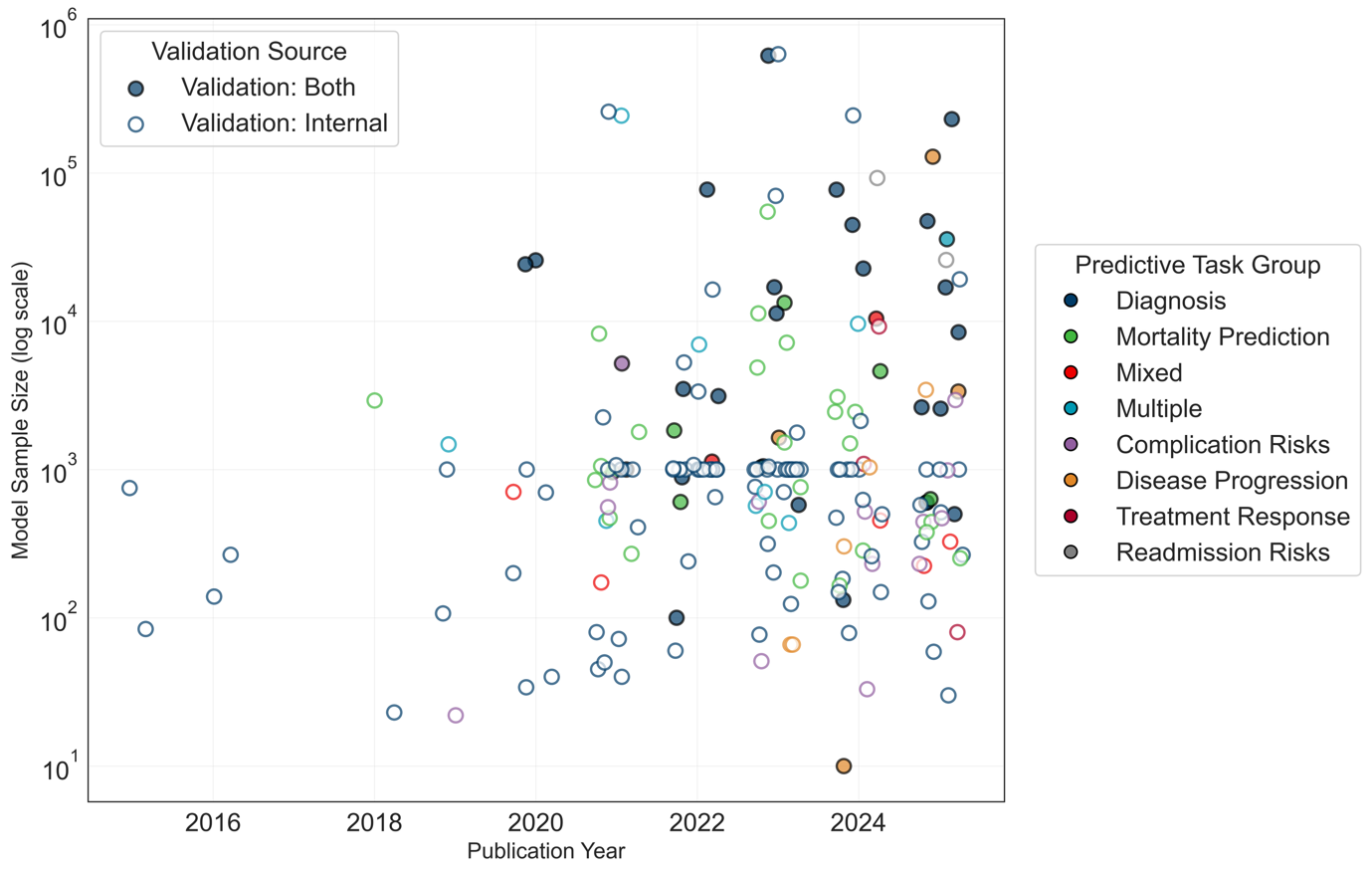


Supplementary Figure 3 | Cohort sample growth and validation of the included studies.


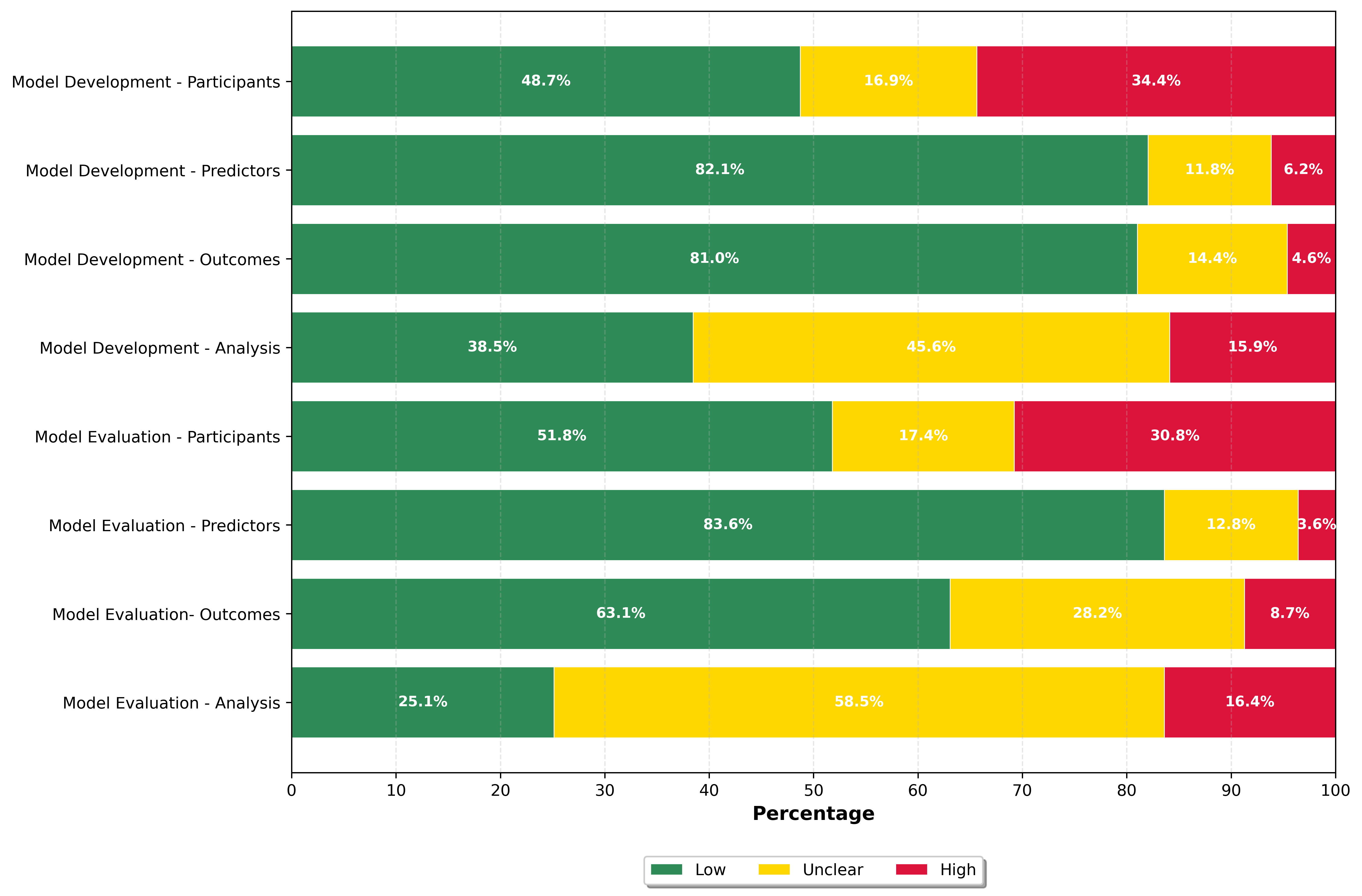


Supplementary Figure 4 | Summary risk of bias of included studies.

Supplementary Table 1: Detailed characteristics of included studies

| **Number** | **reference** | **DOI** | **vhd_type_group** | **mixed_vhd_type** | **predictive_task_group** | **mixed_predictive_task** | **modality** | **multimodal_data_fusion** | **data_source** | **multimodal_type** | **model_architecture** | **model_sample_size** | **study_setting** | **study_design** | **performance_metrics** | **validation_source** | **validation_type** | **post_hoc_explainability** | **model_reproducibility** | **data_labelling** | **technology_readiness_level** | **publication_year** | **more_models_reported** | **model_comparison** | **better_performance** |
| --- | --- | --- | --- | --- | --- | --- | --- | --- | --- | --- | --- | --- | --- | --- | --- | --- | --- | --- | --- | --- | --- | --- | --- | --- | --- |
| **1** | Long et al. | 10.1161/CIRCULATIONAHA.124.068996 | Mitral Regurgitation |  | Diagnosis |  | Single |  | Echocardiography |  | CNN Based Model | 44628 | Multicenter | Retrospective | Mixed | Both | Holdout Validation + Independent | None | None | Expert adjudication | 5 | **2024** | **Yes** | **Yes, compared with cardiologist diagnosis** | **Yes** |
| 2 | Hausleiter et al. | 10.1093/eurheartj/ehad871 | Mitral Regurgitation |  | Mortality Prediction |  | Single |  | Tabular Clinical Data |  | Tree Based Model | 4600 | Multicenter | Retrospective | Mixed | Both | K-fold cross validation + Independent | SHapley Additive exPlanations | None | External Linkage Outcome | 5 | 2024 | No | Yes, compared with existing standard | Yes |
| 3 | Ding et al. | 10.1155/2023/7382316 | Mixed | MS, MR, AS, MVP | Diagnosis |  | Single |  | Heart Sound |  | CNN Based Model | 1000 | Not Specified | Retrospective | Mixed | Internal | Holdout Validation | None | Partial | Proxy Labels | 3 & 4 | 2023 | Yes | No comparison reported |  |
| 4 | Vrudhula et al. | 10.1161/CIRCULATIONAHA.124.069047 | Mitral Regurgitation |  | Diagnosis |  | Single |  | Echocardiography |  | CNN Based Model | 22661 | Single-center | Retrospective | Mixed | Both | Holdout Validation + Independent | Saliency Map | Partial | Expert adjudication | 5 | 2024 | No | No comparison reported |  |
| 5 | Kwiecinski et al. | 10.1093/ehjqcco/qcad002 | Aortic Stenosis |  | Mortality Prediction |  | Single |  | Tabular Clinical Data |  | Tree Based Model | 604 | Single-center | Retrospective | Mixed | Both | K-fold cross validation + Independent | Feature Importance | None | External Linkage Outcome | 5 | 2022 | No | Yes, compared with existing standard | Yes |
| 6 | Anand et al. | 10.1093/ehjdh/ztad006 | Aortic Regurgitation |  | Mortality Prediction |  | Single |  | Tabular Clinical Data |  | Tree Based Model | 1035 | Single-center | Retrospective | Mixed | Both | K-fold cross validation + Independent | Feature Importance | None | External Linkage Outcome | 5 | 2023 | Yes | No comparison reported |  |
| 7 | Hernandez-Suarez et al. | 10.1016/j.carrev.2020.06.017 | Mitral Regurgitation |  | Mortality Prediction |  | Single |  | Tabular Clinical Data |  | Probabilistic Based Model | 849 | Multicenter | Retrospective | AUROC | Internal | Holdout Validation | None | None | Diagnosis/Procedure Codes | 3 & 4 | 2021 | Yes | No comparison reported |  |
| 8 | Shimoni et al. | 10.1016/j.jacadv.2024.101135 | Aortic Stenosis |  | Mixed | Prognosis, Mortality Prediction | Single |  | Tabular Clinical Data |  | Tree Based Model | 10407 | Single-center | Retrospective | Mixed | Both | Holdout Validation + Independent | SHapley Additive exPlanations | None | External Linkage Outcome | 5 | 2024 | No | No comparison reported |  |
| 9 | Kilic et al. | 10.1016/j.athoracsur.2020.05.107 | Aortic Stenosis |  | Multiple |  | Single |  | Tabular Clinical Data |  | Tree Based Model | 243142 | Multicenter | Retrospective | Mixed | Internal | Holdout Validation | None | None | Registry/Trial Endpoints | 3 & 4 | 2021 | No | Yes, compared with existing standard | Yes |
| 10 | Hata et al. | 10.1109/EMBC44109.2020.9175151 | Aortic Stenosis |  | Diagnosis |  | Single |  | Electrocardiogram |  | CNN Based Model | 700 | Single-center | Retrospective | Mixed | Internal | Holdout Validation | GRAD-CAM | None | Expert adjudication | 3 & 4 | 2020 | No | No comparison reported |  |
| 11 | Jamil et al. | 10.1016/j.compbiomed.2023.106734 | Mixed | MS, MR, AS, MVP | Diagnosis |  | Single |  | Heart Sound |  | Transformer Based Model | 1000 | Not Specified | Retrospective | Mixed | Internal | K-fold cross validation + Holdout Validation | None | Partial | Proxy Labels | 3 & 4 | 2023 | Yes | No comparison reported |  |
| 12 | Theis et al. | 10.1016/j.ejrad.2023.111150 | Aortic Stenosis |  | Mortality Prediction |  | Single |  | Computed Tomography |  | CNN Based Model | 760 | Single-center | Retrospective | Mixed | Internal | K-fold cross validation + Holdout Validation | None | None | Expert adjudication | 3 & 4 | 2023 | Yes | No comparison reported |  |
| 13 | Alkhodari & Fraiwan | 10.1016/j.cmpb.2021.105940 | Mixed | MS, MR, AS, MVP | Diagnosis |  | Single |  | Heart Sound |  | Ensemble / Hybrid Model | 1000 | Not Specified | Retrospective | Mixed | Internal | K-fold cross validation | None | Partial | Proxy Labels | 3 & 4 | 2021 | Yes | No comparison reported |  |
| 14 | Yang et al. | 10.1016/j.jcmg.2021.08.015 | Multiple | MS, AS, AR | Diagnosis |  | Single |  | Echocardiography |  | Ensemble / Hybrid Model | 3491 | Multicenter | Ambispective | Mixed | Both | Holdout Validation + Independent | None | Partial | Proxy Labels | 3 & 4 | 2022 | No | No comparison reported |  |
| 15 | Agasthi et al. | 10.1016/j.carrev.2020.08.010 | Aortic Stenosis |  | Mortality Prediction |  | Single |  | Tabular Clinical Data |  | Tree Based Model | 1055 | Multicenter | Retrospective | AUROC | Internal | K-Fold Cross Validation | Feature Importance | None | Expert adjudication | 3 & 4 | 2021 | No | Yes, compared with existing standard | Yes |
| 16 | Kachroo et al. | 10.1111/jocs.16060 | Mitral Regurgitation |  | Mixed | Prognosis, Mortality Prediction | Single |  | Tabular Clinical Data |  | Classical Machine Learning | 173 | Single-center | Retrospective | Mixed | Internal | Holdout Validation | Feature Importance | None | Mixed | 3 & 4 | 2021 | Yes | No comparison reported |  |
| 17 | Vaid et al. | 10.1038/s43856-023-00240-w | Multiple | AS, MR | Diagnosis |  | Multimodal | Intermediate | Multimodal | Tabular Clinical Data + Electrocardiogram | Ensemble / Hybrid Model | 617338 | Multicenter | Retrospective | Mixed | Both | K-fold cross validation + Independent | Backprop-based Feature Attribution | Partial | NLP-Derived Labels | 3 & 4 | 2023 | Yes | No comparison reported |  |
| 18 | Cheng et al. | 10.1161/JAHA.121.024168 | Aortic Regurgitation |  | Diagnosis |  | Single |  | Echocardiography |  | CNN Based Model | 3354 | Single-center | Retrospective | Mixed | Internal | Holdout Validation | Backprop-based Feature Attribution | Partial | Expert adjudication | 3 & 4 | 2022 | No | No comparison reported |  |
| 19 | Cruz et al | 10.1016/j.surg.2024.07.011 | Mitral Regurgitation |  | Mortality Prediction |  | Single |  | Tabular Clinical Data |  | Probabilistic Based Model | 2447 | Multicenter | Retrospective | Mixed | Internal | K-fold cross validation + Holdout Validation | SHapley Additive exPlanations | None | Diagnosis/Procedure Codes | 3 & 4 | 2024 | Yes | No comparison reported |  |
| 20 | Gomes et al. | 10.1007/s00392-020-01691-0 | Aortic Stenosis |  | Multiple | Mortality Prediction, Complication Risks | Single |  | Tabular Clinical Data |  | Tree Based Model | 451 | Single-center | Retrospective | Mixed | Internal | K-Fold Cross Validation | Feature Importance | Partial | Expert adjudication | 3 & 4 | 2021 | Yes | Yes, compared with existing standard | Yes |
| 21 | Navarese et al. | 10.1016/j.jcin.2021.03.024 | Aortic Stenosis |  | Complication Risks |  | Single |  | Tabular Clinical Data |  | Classical Machine Learning | 5185 | Multicenter | Prospective | Mixed | Both | Bootstrap + Independent | None | None | Expert adjudication | 3 & 4 | 2021 | No | Yes, compared with existing standard | Yes |
| 22 | Castela Forte et al. | 10.1038/s41598-021-82403-0 | Multiple | AR, AS, MR, MS | Mortality Prediction |  | Single |  | Tabular Clinical Data |  | Ensemble / Hybrid Model | 8241 | Single-center | Retrospective | Mixed | Internal | K-Fold Cross Validation | Feature Importance | None | External Linkage Outcome | 3 & 4 | 2021 | Yes | No comparison reported |  |
| 23 | Huang et al. | 10.3390/bioengineering10111319 | Mixed | MR, TR | Diagnosis |  | Single |  | Echocardiography |  | CNN Based Model | 315 | Single-center | Retrospective | Mixed | Internal | Holdout Validation | None | None | Imaging/Signal Annotation Labels | 3 & 4 | 2023 | Yes | No comparison reported |  |
| 24 | Penso et al. | 10.3390/ bioengineering8090117 | Mitral Regurgitation |  | Complication Risks |  | Single |  | Tabular Clinical Data |  | Tree Based Model | 817 | Single-center | Retrospective | Mixed | Internal | Holdout Validation | SHapley Additive exPlanations | None | Imaging/Signal Annotation Labels | 3 & 4 | 2021 | Yes | No comparison reported |  |
| 25 | Roy et al. | 10.1007/s11517-023-02827-w | Mixed | MS, AS, MR, AR, MVP | Diagnosis |  | Single |  | Heart Sound |  | CNN Based Model | 70000 | Not Specified | Retrospective | Mixed | Internal | Holdout Validation | None | Partial | Proxy Labels | 3 & 4 | 2023 | Yes | No comparison reported |  |
| 26 | Zhu et al. | 10.1016/j.hjc.2024.04.003 | Mitral Regurgitation |  | Complication Risks |  | Single |  | Tabular Clinical Data |  | Classical Machine Learning | 231 | Single-center | Retrospective | Mixed | Internal | K-fold cross validation + Holdout Validation | None | None | Imaging/Signal Annotation Labels | 3 & 4 | 2024 | Yes | No comparison reported |  |
| 27 | Sanabria et al. | 10.1016/j.jacadv.2024.101234 | Aortic Stenosis |  | Disease Progression |  | Single |  | Tabular Clinical Data |  | Tree Based Model | 303 | Single-center | Prospective | Mixed | Internal | Holdout Validation | None | None | Mixed | 3 & 4 | 2024 | Yes | No comparison reported |  |
| 28 | Ma et al. | 10.1016/j.cmpb.2023.107906 | Mixed | MS, AS, MR, AR, MVP | Diagnosis |  | Single |  | Heart Sound |  | CNN Based Model | 1000 | Not Specified | Retrospective | Mixed | Internal | K-fold cross validation | GRAD-CAM | Partial | Proxy Labels | 3 & 4 | 2024 | Yes | No comparison reported |  |
| 29 | Zisiopoulou et al. | 10.1136/ openhrt-2023-002540 | Aortic Stenosis |  | Mortality Prediction |  | Single |  | Tabular Clinical Data |  | Classical Machine Learning | 284 | Single-center | Prospective | Mixed | Internal | Holdout Validation | None | None | Proxy Labels | 3 & 4 | 2024 | Yes | Yes, compared with existing standard | Yes |
| 30 | Tison et al. | 10.1016/j.jacadv.2023.100446 | Mitral Valve Prolapse |  | Multiple | Mortality Prediction, Complication Risks | Single |  | Electrocardiogram |  | CNN Based Model | 569 | Single-center | Retrospective | Mixed | Internal | Holdout Validation | Custom | None | Mixed | 3 & 4 | 2023 | No | No comparison reported |  |
| 31 | Dasi et al. | 10.1016/j.xjtc.2023.11.011 | Aortic Stenosis |  | Treatment Response |  | Single |  | Tabular Clinical Data |  | Ensemble / Hybrid Model | 1091 | Single-center | Retrospective | Others | Internal | Holdout Validation | Feature Importance | None | Imaging/Signal Annotation Labels | 3 & 4 | 2024 | Yes | No comparison reported |  |
| 32 | Kim et al. | 10.1088/1361-6560/ad22a4 | Aortic Regurgitation |  | Diagnosis |  | Single |  | Echocardiography |  | Transformer Based Model | 183 | Single-center | Retrospective | Mixed | Internal | Holdout Validation | None | None | Expert adjudication | 3 & 4 | 2024 | Yes | No comparison reported |  |
| 33 | Barbieri et al. | 10.3390/jcm13133691 | Aortic Stenosis |  | Mortality Prediction |  | Single |  | Tabular Clinical Data |  | Feedforward / ANN | 3079 | Multicenter | Retrospective | AUROC | Internal | Holdout Validation | None | None | External Linkage Outcome | 3 & 4 | 2024 | No | Yes, compared with existing standard | Yes |
| 34 | Erdogan et al. | 10.14744/SEMB.2024.00836 | Aortic Stenosis |  | Mixed | Mortality Prediction, Complication Risks | Single |  | Tabular Clinical Data |  | Tree Based Model | 453 | Multicenter | Retrospective | Mixed | Internal | Holdout Validation | SHapley Additive exPlanations | None | Registry/Trial Endpoints | 3 & 4 | 2024 | Yes | No comparison reported |  |
| 35 | Lopes et al. | 10.3389/fcvm.2021.787246 | Aortic Stenosis |  | Mortality Prediction |  | Single |  | Tabular Clinical Data |  | Feedforward / ANN | 1791 | Multicenter | Retrospective | AUROC | Internal | K-fold cross validation | None | None | External Linkage Outcome | 3 & 4 | 2021 | Yes | No comparison reported |  |
| 36 | Mamprin et al. | 10.3390/bioengineering8020022 | Aortic Stenosis |  | Mortality Prediction |  | Single |  | Tabular Clinical Data |  | Tree Based Model | 270 | Single-center | Retrospective | Mixed | Internal | Holdout Validation | SHapley Additive exPlanations | None | External Linkage Outcome | 3 & 4 | 2021 | Yes | Yes, compared with existing standard | Yes |
| 37 | Ribeiro et al. | 10.3390/bioengineering11010058 | Mixed |  | Diagnosis |  | Single |  | Electrocardiogram |  | Tree Based Model | 79 | Single-center | Retrospective | Mixed | Internal | leave-one-out cross-validation | None | Partial | Imaging/Signal Annotation Labels | 3 & 4 | 2024 | Yes | No comparison reported |  |
| 38 | Lopes et al. | 10.1016/j.heliyon.2023.e17139 | Aortic Stenosis |  | Mortality Prediction |  | Single |  | Tabular Clinical Data |  | Classical Machine Learning | 11291 | Multicenter | Retrospective | Mixed | Internal | K-fold cross validation | None | None | Registry/Trial Endpoints | 3 & 4 | 2023 | Yes | No comparison reported |  |
| 39 | Zhu et al. | 10.3390/jcdd10020087 | Mixed | As, AR, MS, MR, TS, TR, PS, PR | Mortality Prediction |  | Single |  | Tabular Clinical Data |  | Classical Machine Learning | 7163 | Multicenter | Retrospective | Mixed | Internal | K-fold cross validation + Holdout Validation | None | None | Registry/Trial Endpoints | 3 & 4 | 2023 | Yes | Yes, compared with existing standard | Yes |
| 40 | Suboh et al. | 10.1063/1.4926642 | Mixed | MR,MS,AR,AS, | Diagnosis |  | Single |  | Heart Sound |  | Classical Machine Learning | 750 | Single-center | Retrospective | Accuracy | Internal | Monte Carlo cross-validation | None | None | Imaging/Signal Annotation Labels | 3 & 4 | 2015 | Yes | No comparison reported |  |
| 41 | Das et al. | 10.1109/INDICON56171.2022.10039701 | Mixed | MS, AS, MR, AR, MVP | Diagnosis |  | Single |  | Heart Sound |  | CNN Based Model | 1000 | Not Specified | Retrospective | Mixed | Internal | K-fold cross validation | None | Partial | Proxy Labels | 3 & 4 | 2022 | Yes | No comparison reported |  |
| 42 | Chang et al. | 10.1109/ISOCC59558.2023.10396555 | Mixed | MS, AS, MR, AR, MVP | Diagnosis |  | Single |  | Heart Sound |  | CNN Based Model | 1000 | Not Specified | Retrospective | Accuracy | Internal | K-fold cross validation | None | Partial | Proxy Labels | 3 & 4 | 2023 | No | No comparison reported |  |
| 43 | Joukhadar et al. | 10.1080/23311916.2020.1856757 | Mixed | MS,MR,AS,AR,TR,TS, PS,PR | Diagnosis |  | Single |  | Heart Sound |  | Feedforward / ANN | 200 | Not Specified | Retrospective | Mixed | Internal | Holdout Validation | None | None | Proxy Labels | 3 & 4 | 2020 | Yes | No comparison reported |  |
| 44 | Flores-Alonso et al. | 10.3390/app12083780 | Mixed | MS, AS, MR, AR, MVP | Diagnosis |  | Single |  | Heart Sound |  | Ensemble / Hybrid Model | 1000 | Not Specified | Retrospective | Mixed | Internal | Holdout Validation | None | Partial | Proxy Labels | 3 & 4 | 2022 | Yes | No comparison reported |  |
| 45 | Yildirim | 10.1002/cpe.7232 | Mixed | MS, AS, MR, AR, MVP | Diagnosis |  | Single |  | Heart Sound |  | CNN Based Model | 1000 | Not Specified | Retrospective | Mixed | Internal | Holdout Validation | None | Partial | Proxy Labels | 3 & 4 | 2022 | Yes | No comparison reported |  |
| 46 | Maity et al. | 10.1016/j.bspc.2023.104805 | Mixed | MS, AS, MR, AR, MVP | Diagnosis |  | Single |  | Heart Sound |  | CNN Based Model | 1000 | Not Specified | Retrospective | Mixed | Internal | K-fold cross validation | None | Partial | Proxy Labels | 3 & 4 | 2023 | Yes | No comparison reported |  |
| 47 | Oh et al. | 10.1016/j.cmpb.2020.105604 | Mixed | MS, AS, MR, AR, MVP | Diagnosis |  | Single |  | Heart Sound |  | CNN Based Model | 1000 | Not Specified | Retrospective | Mixed | Internal | K-fold cross validation | None | Partial | Proxy Labels | 3 & 4 | 2020 | No | No comparison reported |  |
| 48 | Karhade et al. | 10.1109/TIM.2022.3163156 | Mixed | MS, AS, MR, AR, MVP | Diagnosis |  | Single |  | Heart Sound |  | CNN Based Model | 1000 | Not Specified | Retrospective | Mixed | Internal | Leave-One-Out Cross-Validation | None | Partial | Proxy Labels | 3 & 4 | 2022 | Yes | No comparison reported |  |
| 49 | Elnaggar et al. | 10.23919/CinC53138.2021.9662695 | Aortic Stenosis |  | Diagnosis |  | Multimodal | Early | Multimodal | Seismocardiography + Gyrocardiography | CNN Based Model | 72 | Multicenter | Prospective | Mixed | Internal | Leave-subject-out Cross validation | None | None | Proxy Labels | 3 & 4 | 2021 | No | No comparison reported |  |
| 50 | Tartarisco et al. | 10.1016/j.eswa.2023.121772 | Mixed | AS,AR,MS, MR | Diagnosis |  | Single |  | Heart Sound |  | RNN Based | 132 | Multicenter | Prospective | Mixed | Both | K-fold cross validation + Independent | None | None | Imaging/Signal Annotation Labels | 3 & 4 | 2024 | Yes | No comparison reported |  |
| 51 | Roy et al. | 10.1016/j.bspc.2023.105086 | Mixed | MS, AS, MR, AR, MVP | Diagnosis |  | Single |  | Heart Sound |  | CNN Based Model | 1000 | Not Specified | Retrospective | Mixed | Internal | Holdout Validation | None | Partial | Proxy Labels | 3 & 4 | 2023 | No | No comparison reported |  |
| 52 | Bhardwaj et al. | 10.1109/TIM.2023.3274174 | Mixed | MS, AS, MR, AR, MVP | Diagnosis |  | Single |  | Heart Sound |  | CNN Based Model | 1000 | Not Specified | Retrospective | Mixed | Internal | K-fold cross validation + Leave-One-Out Cross-Validation | Mixed | Partial | Proxy Labels | 3 & 4 | 2023 | No | No comparison reported |  |
| 53 | Chowdhury et al. | 10.1109/JSEN.2022.3196263 | Mixed | MS, AS, MR, AR, MVP | Diagnosis |  | Single |  | Heart Sound |  | CNN Based Model | 3126 | Not Specified | Retrospective | Mixed | Both | Holdout Validation + Independent | None | Partial | Proxy Labels | 3 & 4 | 2022 | No | No comparison reported |  |
| 54 | Vafaeezadeh et al. | 10.1002/ima.22885 | Mixed | MS, MVP | Diagnosis |  | Single |  | Echocardiography |  | Ensemble / Hybrid Model | 1773 | Single-center | Retrospective | Mixed | Internal | Holdout Validation | GRAD-CAM | Partial | Expert adjudication | 3 & 4 | 2023 | Yes | No comparison reported |  |
| 55 | Tuncer et al. | 10.1016/j.ins.2021.01.088 | Mixed | MS, AS, MR, AR, MVP | Diagnosis |  | Single |  | Heart Sound |  | Classical Machine Learning | 1000 | Not Specified | Retrospective | Mixed | Internal | K-fold cross validation | None | Partial | Proxy Labels | 3 & 4 | 2021 | Yes | No comparison reported |  |
| 56 | Mani et al. | 10.4103/jicc.jicc_44_23 | Mitral Stenosis |  | Treatment Response |  | Single |  | Tabular Clinical Data |  | Tree Based Model | 9212 | Single-center | Retrospective | Mixed | Internal | K-fold cross validation | SHapley Additive exPlanations | None | Proxy Labels | 3 & 4 | 2024 | Yes | No comparison reported |  |
| 57 | Hafiz et al. | 10.1504/IJMEI.2021.115970 | Mixed | MS, PS | Diagnosis |  | Single |  | Ambient Sensors |  | Classical Machine Learning | 80 | Multicenter | Prospective | Mixed | Internal | Holdout Validation | None | None | Expert adjudication | 3 & 4 | 2021 | Yes | No comparison reported |  |
| 58 | Yu et al. | 10.2147/JIR.S453100 | Aortic Stenosis |  | Disease Progression |  | Single |  | Genomic Data |  | Classical Machine Learning | 10 | Single-center | Retrospective | AUROC | Both | Holdout Validation + Independent | None | None | Mixed | 5 | 2024 | No | No comparison reported |  |
| 59 | Lin et al. | 10.18632/aging.205835 | Multiple | AS, AR, PR, TR, MR | Diagnosis |  | Single |  | Electrocardiogram |  | CNN Based Model | 77047 | Single-center | Retrospective | Mixed | Both | Holdout Validation + Independent | None | None | Imaging/Signal Annotation Labels | 3 & 4 | 2024 | No | No comparison reported |  |
| 60 | Brüggemann et al. | 10.1038/s41598-024-63022-x | Aortic Stenosis |  | Mortality Prediction |  | Multimodal | Intermediate | Multimodal | Tabular Clinical Data + CT | Ensemble / Hybrid Model | 1499 | Single-center | Retrospective | AUROC | Internal | K-fold cross validation | None | None | Registry/Trial Endpoints | 3 & 4 | 2024 | Yes | Yes, compared with existing standard | Yes |
| 61 | Kalmady et al. | 10.1038/s41746-024-01130-8 | Multiple | AS, MVP, MS | Diagnosis |  | Single |  | Electrocardiogram |  | CNN Based Model | 244077 | Multicenter | Retrospective | Mixed | Internal | K-fold cross validation + Leave-One-Out Cross-Validation | GRAD-CAM | Partial | Diagnosis/Procedure Codes | 3 & 4 | 2024 | Yes | No comparison reported |  |
| 62 | Al-Tam et al. | 10.1109/ACCESS.2024.3451660 | Mixed | MS, AS, MR, AR, MVP | Diagnosis |  | Single |  | Heart Sound |  | Ensemble / Hybrid Model | 1000 | Not Specified | Retrospective | Mixed | Internal | K-fold cross validation | None | Partial | Proxy Labels | 3 & 4 | 2024 | Yes | No comparison reported |  |
| 63 | Yang et al. | 10.1109/TBME.2019.2942741 | Aortic Stenosis |  | Diagnosis |  | Multimodal | Early | Multimodal | Seismocardiography + Gyrocardiography | Tree Based Model | 40 | Multicenter | Prospective | Mixed | Internal | Holdout Validation | None | None | Proxy Labels | 3 & 4 | 2020 | Yes | No comparison reported |  |
| 64 | Jumphoo et al. | 10.1109/ACCESS.2024.3357946 | Mixed | MS, AS, MR, AR, MVP | Diagnosis |  | Single |  | Heart Sound |  | Ensemble / Hybrid Model | 1000 | Not Specified | Retrospective | Mixed | Internal | K-fold cross validation | None | Partial | Proxy Labels | 3 & 4 | 2024 | Yes | No comparison reported |  |
| 65 | Morshed et al. | 10.1109/JSEN.2023.3289109 | Mixed | MS, AS, MR, AR, MVP | Diagnosis |  | Single |  | Heart Sound |  | CNN Based Model | 1000 | Not Specified | Retrospective | Mixed | Internal | Holdout Validation | None | Partial | Proxy Labels | 3 & 4 | 2023 | No | No comparison reported |  |
| 66 | Waaler et al. | 10.3389/fcvm.2023.1170804 | Multiple | AS, MR, AR, MS | Diagnosis |  | Multimodal | Late | Multimodal | Tabular Clinical Data + Heart Sound | Ensemble / Hybrid Model | 2124 | Single-center | Prospective | Mixed | Internal | K-fold cross validation | None | None | Imaging/Signal Annotation Labels | 3 & 4 | 2024 | Yes | No comparison reported |  |
| 67 | Su et al. | 10.23919/CCC55666.2022.9901903 | Mixed | MS, AS, MR, AR, MVP | Diagnosis |  | Single |  | Heart Sound |  | CNN Based Model | 1000 | Not Specified | Retrospective | Mixed | Internal | K-fold cross validation | None | Partial | Proxy Labels | 3 & 4 | 2022 | No | No comparison reported |  |
| 68 | Khan et al. | 10.1016/j.bspc.2021.103445 | Mixed | MS, AS, MR, AR, MVP | Diagnosis |  | Single |  | Heart Sound |  | Tree Based Model | 1000 | Not Specified | Retrospective | Mixed | Internal | K-fold cross validation | None | Partial | Proxy Labels | 3 & 4 | 2022 | Yes | No comparison reported |  |
| 69 | Ghanayim et al. | 10.1016/j.amjmed.2022.04.032 | Aortic Stenosis |  | Diagnosis |  | Single |  | Heart Sound |  | Classical Machine Learning | 100 | Single-center | Prospective | Mixed | Both | Holdout Validation + Independent | None | None | Imaging/Signal Annotation Labels | 3 & 4 | 2022 | No | No comparison reported |  |
| 70 | Jiang et al. | 10.1016/j.ijcha.2024.101368 | Mixed | MS,MR,TR,AS,AR | Diagnosis |  | Single |  | Heart Sound |  | CNN Based Model | 499 | Multicenter | Prospective | Mixed | Internal | Holdout Validation | None | None | Imaging/Signal Annotation Labels | 3 & 4 | 2024 | No | No comparison reported |  |
| 71 | Hassanien et al. | 10.1093/jigpal/jzv009 | Mixed | AS, MR, AR, MS | Diagnosis |  | Single |  | Heart Sound |  | Feedforward / ANN | 84 | Not Specified | Not Specified | Accuracy | Internal | K-fold cross validation | None | None | Imaging/Signal Annotation Labels | 3 & 4 | 2015 | Yes | No comparison reported |  |
| 72 | Shiraga et al. | 10.3390/s23249834 | Multiple | AS, MR | Diagnosis |  | Multimodal | Late | Multimodal | Heart Sound + Electrocardiogram | Ensemble / Hybrid Model | 1051 | Single-center | Ambispective | Mixed | Both | K-fold cross validation + Independent | None | None | Imaging/Signal Annotation Labels | 3 & 4 | 2023 | Yes | No comparison reported |  |
| 73 | Al-Issa & Alqudah | 10.1038/s41598-022-18293-7 | Mixed | MS, AS, MR, AR, MVP | Diagnosis |  | Single |  | Heart Sound |  | Ensemble / Hybrid Model | 1000 | Not Specified | Retrospective | Mixed | Internal | K-fold cross validation | None | Partial | Proxy Labels | 3 & 4 | 2022 | Yes | No comparison reported |  |
| 74 | Zeng et al. | 10.1007/s10462-022-10184-7 | Mixed | MS, AS, MR, AR, MVP | Diagnosis |  | Single |  | Heart Sound |  | CNN Based Model | 1000 | Not Specified | Retrospective | Mixed | Internal | K-fold cross validation | None | Partial | Proxy Labels | 3 & 4 | 2022 | No | No comparison reported |  |
| 75 | Elias et al. | 10.1016/j.jacc.2022.05.029 | Mixed | AS, AR, MR | Diagnosis |  | Single |  | Electrocardiogram |  | CNN Based Model | 77163 | Multicenter | Retrospective | Mixed | Both | Holdout Validation + Independent | SHapley Additive exPlanations | None | Imaging/Signal Annotation Labels | 3 & 4 | 2022 | No | No comparison reported |  |
| 76 | Gaye et al. | 10.1136/bmjopen-2022-067977 | Mixed | AS, AR | Disease Progression |  | Single |  | Tabular Clinical Data |  | Tree Based Model | 1034 | Single-center | Prospective | AUROC | Internal | K-fold cross validation | None | None | Imaging/Signal Annotation Labels | 3 & 4 | 2024 | Yes | No comparison reported |  |
| 77 | Avola et al. | 10.1016/j.cmpb.2024.108037 | Aortic Stenosis |  | Diagnosis |  | Single |  | Echocardiography |  | Ensemble / Hybrid Model | 260 | Single-center | Retrospective | Mixed | Internal | Holdout Validation | None | Partial | Expert adjudication | 3 & 4 | 2024 | Yes | No comparison reported |  |
| 78 | Li et al. | 10.1002/adts.202300549 | Mixed | MS, AS, MR, AR, MVP | Diagnosis |  | Single |  | Heart Sound |  | CNN Based Model | 1000 | Not Specified | Retrospective | Mixed | Internal | Holdout Validation | None | Partial | Proxy Labels | 3 & 4 | 2024 | No | No comparison reported |  |
| 79 | Penso et al. | 10.3390/jcdd8040044 | Aortic Stenosis |  | Mortality Prediction |  | Single |  | Tabular Clinical Data |  | Feedforward / ANN | 471 | Single-center | Retrospective | Mixed | Internal | K-fold cross validation + Holdout Validation | Mixed | Partial | Expert adjudication | 3 & 4 | 2021 | Yes | Yes, compared with existing standard | Yes |
| 80 | Zhang et al. | 10.1007/s00034-023-02588-9 | Mixed | MS, AS, MR, AR, MVP | Diagnosis |  | Single |  | Heart Sound |  | CNN Based Model | 1000 | Not Specified | Retrospective | Accuracy | Internal | Holdout Validation | None | Partial | Proxy Labels | 3 & 4 | 2024 | No | No comparison reported |  |
| 81 | Shuvo et al. | 10.1016/j.bspc.2023.105272 | Mixed | MS, AS, MR, AR, MVP | Diagnosis |  | Single |  | Heart Sound |  | Ensemble / Hybrid Model | 1000 | Not Specified | Retrospective | Mixed | Internal | K-fold cross validation | None | Partial | Proxy Labels | 3 & 4 | 2023 | Yes | No comparison reported |  |
| 82 | Xiao et al. | 10.1007/978-3-031-43990-2_23 | Mitral Regurgitation |  | Diagnosis |  | Single |  | MRI |  | Ensemble / Hybrid Model | 704 | Multicenter | Retrospective | Mixed | Internal | Holdout Validation | None | None | Expert adjudication | 3 & 4 | 2023 | Yes | No comparison reported |  |
| 83 | Dubey et al. | 10.1109/ICUMT48472.2019.8970870 | Mixed | MS, AS, MR, AR, MVP | Diagnosis |  | Single |  | Heart Sound |  | Classical Machine Learning | 1000 | Not Specified | Retrospective | Mixed | Internal | Holdout Validation | None | Partial | Proxy Labels | 3 & 4 | 2019 | No | No comparison reported |  |
| 84 | Roy et al. | 10.1109/OJIM.2023.3320765 | Mixed | MS, AS, MR, AR, MVP | Diagnosis |  | Single |  | Heart Sound |  | Ensemble / Hybrid Model | 1000 | Not Specified | Retrospective | Mixed | Internal | K-fold cross validation | None | Partial | Proxy Labels | 3 & 4 | 2023 | Yes | No comparison reported |  |
| 85 | Qi et al. | 10.1016/j.bspc.2023.105265 | Mitral Regurgitation |  | Diagnosis |  | Multimodal | Intermediate | Multimodal | Heart Sound + Electrocardiogram | Ensemble / Hybrid Model | 1046 | Single-center | Retrospective | Mixed | Internal | K-fold cross validation | None | None | Expert adjudication | 3 & 4 | 2023 | Yes | No comparison reported |  |
| 86 | Zeng et al. | 10.1007/s10462-021-09969-z | Mixed | MS, AS, MR, AR, MVP | Diagnosis |  | Single |  | Heart Sound |  | Feedforward / ANN | 1000 | Not Specified | Retrospective | Mixed | Internal | K-fold cross validation | None | Partial | Proxy Labels | 3 & 4 | 2021 | No | No comparison reported |  |
| 87 | Kang et al. | 10.1093/icvts/ivad176 | Mitral Regurgitation |  | Multiple | Mortality Prediction, Trearment Response | Single |  | Tabular Clinical Data |  | Tree Based Model | 436 | Single-center | Retrospective | C-index | Internal | K-fold cross validation | Feature Importance | None | Mixed | 3 & 4 | 2023 | Yes | No comparison reported |  |
| 88 | Deb et al. | 10.1136/openhrt-2023-002417 | Tricuspid Regurgitation |  | Mortality Prediction |  | Single |  | Tabular Clinical Data |  | Tree Based Model | 13312 | Single-center | Retrospective | C-index | Both | K-fold cross validation+ Independent | Feature Importance | None | External Linkage Outcome | 3 & 4 | 2023 | Yes | No comparison reported |  |
| 89 | Zahid et al. | 10.1016/j.cpcardiol.2023.102143 | Aortic Stenosis |  | Readmission Risks |  | Single |  | Tabular Clinical Data |  | Ensemble / Hybrid Model | 92363 | Multicenter | Retrospective | Mixed | Internal | Holdout Validation | None | None | Diagnosis/Procedure Codes | 3 & 4 | 2024 | No | No comparison reported |  |
| 90 | Holste et al. | 10.1093/eurheartj/ehad456 | Aortic Stenosis |  | Diagnosis |  | Single |  | Echocardiography |  | Ensemble / Hybrid Model | 11297 | Multicenter | Retrospective | Mixed | Both | Holdout Validation + Independent | Saliency Map | Partial | Expert adjudication | 3 & 4 | 2023 | No | No comparison reported |  |
| 91 | Ebrahimkhani et al. | 10.1007/s10439-023-03342-7 | Aortic Stenosis |  | Diagnosis |  | Multimodal | Intermediate | Multimodal | Seismocardiography + Tabular Clinical Data | Ensemble / Hybrid Model | 77 | Single-center | Prospective | Mixed | Internal | Leave-subject-out cross-validation | None | None | Imaging/Signal Annotation Labels | 3 & 4 | 2023 | No | No comparison reported |  |
| 92 | Nguyen et al. | 10.1007/s00034-022-02124-1 | Mixed | MS, AS, MR, AR, MVP | Diagnosis |  | Single |  | Heart Sound |  | CNN Based Model | 1000 | Not Specified | Retrospective | Mixed | Internal | Holdout Validation | None | Partial | Proxy Labels | 3 & 4 | 2023 | Yes | No comparison reported |  |
| 93 | Namasivayam et al. | 10.3389/fcvm.2023.1153814 | Aortic Stenosis |  | Disease Progression |  | Single |  | Echocardiography |  | Feedforward / ANN | 66 | Single-center | Retrospective | Mixed | Internal | Holdout Validation | Feature Importance | None | Imaging/Signal Annotation Labels | 3 & 4 | 2023 | No | No comparison reported |  |
| 94 | Arslan & Karhan | 10.1016/j.jksuci.2021.12.019 | Mixed | MS, AS, MR, AR, MVP | Diagnosis |  | Single |  | Heart Sound |  | Feedforward / ANN | 1000 | Not Specified | Retrospective | Mixed | Internal | 5-Fold cross validation | None | Partial | Proxy Labels | 3 & 4 | 2022 | Yes | No comparison reported |  |
| 95 | Rajeshwari et al. | 10.1109/TIM.2023.3240995 | Mitral Valve Prolapse |  | Diagnosis |  | Single |  | Heart Sound |  | CNN Based Model | 124 | Not Specified | Retrospective | Mixed | Internal | Holdout Validation | GRAD-CAM | Partial | Imaging/Signal Annotation Labels | 3 & 4 | 2023 | Yes | No comparison reported |  |
| 96 | Wahlang et al. | 10.3390/electronics10040495 | Mixed | MR, AR, TR | Diagnosis |  | Single |  | Echocardiography |  | RNN Based | 1070 | Single-center | Retrospective | Mixed | Internal | Holdout Validation | None | None | Imaging/Signal Annotation Labels | 3 & 4 | 2021 | Yes | No comparison reported |  |
| 97 | Sánchez-Puente et al. | 10.1016/j.jcmg.2022.12.008 | Aortic Stenosis |  | Disease Progression |  | Single |  | Tabular Clinical Data |  | Tree Based Model | 1638 | Single-center | Retrospective | Mixed | Both | K-fold cross validation+ Independent | None | None | Imaging/Signal Annotation Labels | 5 | 2023 | Yes | Yes, compared with existing standard | Yes |
| 98 | Ueda et al. | 10.1016/ S2589-7500(23)00107-3 | Mixed | MR, MS, AS, AR, TR, PR | Diagnosis |  | Single |  | Chest X-ray |  | CNN Based Model | 16946 | Multicenter | Retrospective | Mixed | Both | K-fold cross validation+ Independent | Mixed | Partial | Imaging/Signal Annotation Labels | 5 | 2023 | No | No comparison reported |  |
| 99 | Zhou et al. | 10.3389/fcvm.2022.866257 | Mixed | MR, MS | Mortality Prediction |  | Single |  | Tabular Clinical Data |  | Classical Machine Learning | 1833 | Single-center | Retrospective | AUROC | Both | Holdout Validation + Independent | None | None | External Linkage Outcome | 3 & 4 | 2022 | Yes | Yes, compared with existing standard | Yes |
| 100 | Luosang et al. | 10.1007/s10489-023-04877-x | Aortic Stenosis |  | Complication Risks |  | Multimodal | Intermediate | Multimodal | Tabular Clinical Data + Electrocardiogram | Ensemble / Hybrid Model | 606 | Single-center | Retrospective | Mixed | Internal | K-fold cross validation | None | None | Expert adjudication | 3 & 4 | 2023 | Yes | No comparison reported |  |
| 101 | Prabhakar & Won | 10.1016/j.eswa.2023.119720 | Mixed | AR, AS, MR, MVP | Diagnosis |  | Single |  | Heart Sound |  | Classical Machine Learning | 764 | Multicenter | Retrospective | Mixed | Internal | K-fold cross validation | None | Partial | Expert adjudication | 3 & 4 | 2023 | Yes | No comparison reported |  |
| 102 | Kwak et al. | 10.1093/ehjci/jead077 | Mitral Regurgitation |  | Mortality Prediction |  | Single |  | Tabular Clinical Data |  | Tree Based Model | 1521 | Multicenter | Retrospective | C-index | Internal | Holdout Validation | Partial Dependency Plot | None | External Linkage Outcome | 3 & 4 | 2023 | No | Yes, compared with existing standard | Yes |
| 103 | Chen et al. | 10.4103/ACCJ.ACCJ_13_22 | Aortic Stenosis |  | Mortality Prediction |  | Single |  | Tabular Clinical Data |  | Tree Based Model | 450 | Multicenter | Retrospective | Mixed | Internal | K-fold cross validation | None | None | External Linkage Outcome | 3 & 4 | 2023 | Yes | No comparison reported |  |
| 104 | Strange et al. | 10.1136/openhrt-2023-002265 | Aortic Stenosis |  | Diagnosis |  | Single |  | Echocardiography |  | Feedforward / ANN | 631824 | Multicenter | Retrospective | Mixed | Internal | Holdout Validation | None | None | Imaging/Signal Annotation Labels | 3 & 4 | 2023 | No | No comparison reported |  |
| 105 | Kumar et al. | 10.1080/23311916.2018.1502906 | Mixed | AS,AR,PS,MS,MR | Diagnosis |  | Single |  | Heart Sound |  | Feedforward / ANN | 23 | Not Specified | Retrospective | Mixed | Internal | Holdout Validation | None | None | Imaging/Signal Annotation Labels | 3 & 4 | 2018 | No | No comparison reported |  |
| 106 | Mustafić et al. | 10.1007/978-3-030-17971-7_23 | Aortic Stenosis |  | Diagnosis |  | Single |  | Tabular Clinical Data |  | Feedforward / ANN | 107 | Single-center | Retrospective | Others | Internal | Holdout Validation | None | None | Imaging/Signal Annotation Labels | 3 & 4 | 2019 | No | No comparison reported |  |
| 107 | Elvas et al. | 10.3390/electronics12234835 | Aortic Stenosis |  | Diagnosis |  | Single |  | MRI |  | CNN Based Model | 202 | Single-center | Retrospective | Mixed | Internal | Holdout Validation | None | None | Imaging/Signal Annotation Labels | 3 & 4 | 2023 | Yes | No comparison reported |  |
| 108 | Rishal & Satija | 10.1109/INDICON56171.2022.10039770 | Mixed | MS, AS, MR, AR, MVP | Diagnosis |  | Single |  | Heart Sound |  | Tree Based Model | 1000 | Not Specified | Retrospective | Mixed | Internal | Holdout Validation | None | Partial | Proxy Labels | 3 & 4 | 2022 | Yes | No comparison reported |  |
| 109 | Zhou et al. | 10.1016/j.cpcardiol.2022.101464 | Mitral Regurgitation |  | Multiple | Mortality Prediction, Complication Risks | Single |  | Tabular Clinical Data |  | Tree Based Model | 706 | Single-center | Retrospective | Mixed | Internal | K-fold cross validation | Feature Importance | None | External Linkage Outcome | 3 & 4 | 2023 | Yes | No comparison reported |  |
| 110 | Heitzinger et al. | 10.1093/ehjci/jead009 | Tricuspid Regurgitation |  | Mortality Prediction |  | Single |  | Tabular Clinical Data |  | Tree Based Model | 4868 | Single-center | Retrospective | C-index | Internal | Holdout Validation | Feature Importance | None | External Linkage Outcome | 3 & 4 | 2023 | No | No comparison reported |  |
| 111 | Alhwiti et al. | 10.1038/s41598-023-37358-9 | Aortic Stenosis |  | Mortality Prediction |  | Single |  | Tabular Clinical Data |  | Classical Machine Learning | 54739 | Multicenter | Retrospective | Mixed | Internal | Holdout Validation | Feature Importance | Partial | External Linkage Outcome | 3 & 4 | 2023 | Yes | Yes, compared with existing standard | Yes |
| 112 | Zheng et al. | 10.3389/fcvm.2023.1112797 | Mitral Regurgitation |  | Complication Risks |  | Single |  | Tabular Clinical Data |  | Tree Based Model | 51 | Single-center | Prospective | Mixed | Internal | K-fold cross validation | SHapley Additive exPlanations | None | Imaging/Signal Annotation Labels | 3 & 4 | 2023 | Yes | No comparison reported |  |
| 113 | Wessler et al. | 10.1016/j.echo.2023.01.006 | Aortic Stenosis |  | Diagnosis |  | Single |  | Echocardiography |  | CNN Based Model | 577 | Single-center | Retrospective | Mixed | Both | Holdout Validation + Independent | GRAD-CAM | Full | Expert adjudication | 5 | 2023 | No | No comparison reported |  |
| 114 | Asheghan et al. | 10.3389/fcvm.2023.1130152 | Aortic Stenosis |  | Disease Progression |  | Single |  | Computed Tomography |  | Classical Machine Learning | 66 | Single-center | Retrospective | Mixed | Internal | Holdout Validation | None | None | Imaging/Signal Annotation Labels | 3 & 4 | 2023 | Yes | No comparison reported |  |
| 115 | Roy et al. | 10.1016/j.bea.2022.100035 | Mixed | MS, AS, MR, AR, MVP | Diagnosis |  | Single |  | Heart Sound |  | CNN Based Model | 1000 | Not Specified | Retrospective | Mixed | Internal | Holdout Validation | None | Partial | Proxy Labels | 3 & 4 | 2022 | Yes | No comparison reported |  |
| 116 | Barua et al. | 10.1016/j.compbiomed.2022.105599 | Mixed | AS, MS, MR, TR | Diagnosis |  | Single |  | Heart Sound |  | Classical Machine Learning | 651 | Single-center | Prospective | Mixed | Internal | K-fold cross validation | None | Partial | Imaging/Signal Annotation Labels | 3 & 4 | 2022 | No | No comparison reported |  |
| 117 | Talal et al. | 10.1111/exsy.13411 | Mixed | MS, AS, MR, AR, MVP | Diagnosis |  | Single |  | Heart Sound |  | Classical Machine Learning | 1000 | Not Specified | Retrospective | Mixed | Internal | K-fold cross validation | None | Partial | Proxy Labels | 3 & 4 | 2023 | Yes | No comparison reported |  |
| 118 | Makimoto et al. | 10.1093/ehjdh/ztac029 | Aortic Stenosis |  | Diagnosis |  | Single |  | Heart Sound |  | Ensemble / Hybrid Model | 886 | Single-center | Prospective | Mixed | Both | K-fold cross validation+ Independent | GRAD-CAM | None | Imaging/Signal Annotation Labels | 5 | 2022 | Yes | Yes, compared with cardiologist diagnosis | Yes |
| 119 | Roy et al. | 10.1109/ICCECE51049.2023.10085513 | Mixed | MS, AS, MR, AR, MVP | Diagnosis |  | Single |  | Heart Sound |  | CNN Based Model | 1000 | Not Specified | Retrospective | Mixed | Internal | K-fold cross validation | None | Partial | Proxy Labels | 3 & 4 | 2023 | Yes | No comparison reported |  |
| 120 | Bansal et al. | 10.1016/j.carrev.2022.07.024 | Aortic Stenosis |  | Multiple | Mortality Prediction, Complication Risks | Single |  | Tabular Clinical Data |  | Tree Based Model | 6962 | Multicenter | Retrospective | Mixed | Internal | Holdout Validation | Partial Dependency Plot | Partial | External Linkage Outcome | 3 & 4 | 2022 | Yes | No comparison reported |  |
| 121 | Namasivayam et al. | 10. 1136/openhrt-2022-001990 | Aortic Stenosis |  | Mixed | Mortality Prediction, Risk Assessment | Single |  | Tabular Clinical Data |  | Classical Machine Learning | 1130 | Single-center | Retrospective | Mixed | Both | Bootstrapping + Independent | None | None | External Linkage Outcome | 5 | 2022 | Yes | No comparison reported |  |
| 122 | Arslan | 10.1016/j.bspc.2022.103929 | Mixed | MS, AS, MR, AR, MVP | Diagnosis |  | Single |  | Heart Sound |  | Tree Based Model | 1000 | Not Specified | Retrospective | Mixed | Internal | K-fold cross validation | None | Partial | Proxy Labels | 3 & 4 | 2022 | Yes | No comparison reported |  |
| 123 | Voigt et al. | 10.1002/clc.23826 | Aortic Stenosis |  | Diagnosis |  | Single |  | Heart Sound |  | CNN Based Model | 240 | Single-center | Retrospective | Mixed | Internal | K-fold cross validation | None | None | Imaging/Signal Annotation Labels | 3 & 4 | 2022 | No | Yes, compared with cardiologist diagnosis | Yes |
| 124 | Mohammadyari et al. | 10.1038/s41598-024-61685-0 | Aortic Stenosis |  | Mortality Prediction |  | Single |  | Tabular Clinical Data |  | Feedforward / ANN | 165 | Single-center | Retrospective | Mixed | Internal | K-fold cross validation | None | None | Expert adjudication | 3 & 4 | 2024 | Yes | No comparison reported |  |
| 125 | Ueda et al. | 10.1148/ryai.210221 | Mitral Regurgitation |  | Diagnosis |  | Single |  | Chest X-ray |  | CNN Based Model | 5270 | Single-center | Retrospective | Mixed | Internal | Holdout Validation | GRAD-CAM | Partial | Imaging/Signal Annotation Labels | 3 & 4 | 2022 | Yes | No comparison reported |  |
| 126 | Wang et al. | 10.3390/jcdd9030086 | Mixed | MS, AS, MR, AR, MVP | Diagnosis |  | Single |  | Heart Sound |  | CNN Based Model | 1074 | Not Specified | Retrospective | Mixed | Internal | K-fold cross validation | None | Full | Mixed | 3 & 4 | 2022 | Yes | No comparison reported |  |
| 127 | Sawano et al. | 10.1016/j.jjcc.2021.08.029 | Aortic Regurgitation |  | Diagnosis |  | Multimodal | Late | Multimodal | Tabular Clinical Data + Electrocardiogram | Ensemble / Hybrid Model | 16344 | Single-center | Retrospective | Mixed | Internal | Holdout Validation | GRAD-CAM | None | Imaging/Signal Annotation Labels | 3 & 4 | 2022 | Yes | No comparison reported |  |
| 128 | Suhas et al. | 10.1109/INDICON.2016.7839002 | Mixed | AS, MR, MS, AR | Diagnosis |  | Single |  | Heart Sound |  | Ensemble / Hybrid Model | 266 | Not Specified | Retrospective | Mixed | Internal | Cross Validation + Thresholding | None | None | Imaging/Signal Annotation Labels | 3 & 4 | 2016 | Yes | No comparison reported |  |
| 129 | Vimalesvaran et l. | 10.1007/978-3-031-16431-6_54 | Mixed | AS, AR | Diagnosis |  | Single |  | MRI |  | Ensemble / Hybrid Model | 1017 | Multicenter | Retrospective | Mixed | Internal | K-fold cross validation | None | None | NLP-Derived Labels | 3 & 4 | 2022 | Yes | No comparison reported |  |
| 130 | Zhang et al. | 10.1109/ACCESS.2019.2916762 | Aortic Stenosis |  | Complication Risks |  | Single |  | Computed Tomography |  | Classical Machine Learning | 22 | Single-center | Retrospective | Mixed | Internal | Holdout Validation | None | None | Imaging/Signal Annotation Labels | 3 & 4 | 2019 | No | No comparison reported |  |
| 131 | Zweck et al. | 10.1016/j.jcin.2021.06.039 | Mitral Regurgitation |  | Mortality Prediction |  | Single |  | Tabular Clinical Data |  | Tree Based Model | 959 | Multicenter | Retrospective | AUROC | Both | K-fold cross validation+ Independent | Feature Importance | None | External Linkage Outcome | 5 | 2021 | Yes | Yes, compared with existing standard | Yes |
| 132 | Ginsberg et al. | 10.1007/978-3-030-87583-1_20 | Aortic Stenosis |  | Diagnosis |  | Single |  | Echocardiography |  | CNN Based Model | 2247 | Single-center | Retrospective | Mixed | Internal | Holdout Validation | None | None | Imaging/Signal Annotation Labels | 3 & 4 | 2021 | No | No comparison reported |  |
| 133 | Shokouhmand et al. | 10.1038/s41598-021-03441-2 | Aortic Stenosis |  | Diagnosis |  | Multimodal | Early | Multimodal | Seismocardiography + Gyrocardiography + Electrocardiography | Tree Based Model | 45 | Single-center | Prospective | Mixed | Internal | K-fold cross validation + Holdout Validation | SHapley Additive exPlanations | None | Imaging/Signal Annotation Labels | 3 & 4 | 2021 | Yes | No comparison reported |  |
| 134 | Cohen-Shelly et al. | 10.1093/eurheartj/ehab153 | Aortic Stenosis |  | Diagnosis |  | Single |  | Electrocardiogram |  | CNN Based Model | 258607 | Single-center | Retrospective | Mixed | Internal | Holdout Validation | Saliency Map | None | Imaging/Signal Annotation Labels | 3 & 4 | 2021 | No | No comparison reported |  |
| 135 | Kang et al. | 10.3348/kjr.2020.0099 | Aortic Stenosis |  | Diagnosis |  | Single |  | Computed Tomography |  | Tree Based Model | 408 | Single-center | Retrospective | C-index | Internal | Holdout Validation | None | None | Imaging/Signal Annotation Labels | 3 & 4 | 2021 | Yes | No comparison reported |  |
| 136 | Truong et al. | 10.1111/pace.14163 | Aortic Stenosis |  | Complication Risks |  | Single |  | Tabular Clinical Data |  | Tree Based Model | 557 | Single-center | Retrospective | Mixed | Internal | Holdout Validation | Feature Importance | None | Imaging/Signal Annotation Labels | 3 & 4 | 2021 | Yes | No comparison reported |  |
| 137 | Moghaddasi & Nourian | 10.1016/j.compbiomed.2016.03.026 | Mitral Regurgitation |  | Diagnosis |  | Single |  | Echocardiography |  | Classical Machine Learning | 139 | Single-center | Retrospective | Mixed | Internal | K-fold cross validation | None | None | Expert adjudication | 3 & 4 | 2016 | Yes | No comparison reported |  |
| 138 | Shuvo et al. | 10.1109/ACCESS.2021.3063129 | Mixed | MS, AS, MR, AR, MVP | Diagnosis |  | Single |  | Heart Sound |  | Ensemble / Hybrid Model | 1000 | Not Specified | Retrospective | Mixed | Both | K-fold cross validation | None | Partial | Proxy Labels | 5 | 2021 | No | No comparison reported |  |
| 139 | Lopes et al. | 10.1007/s12471-019-1285-7 | Aortic Stenosis |  | Multiple | Mortality Prediction, Prognosis | Single |  | Tabular Clinical Data |  | Multiple | 1478 | Single-center | Retrospective | AUROC | Internal | Monte carlo cross validation | Feature Importance | None | Mixed | 3 & 4 | 2019 | Yes | No comparison reported |  |
| 140 | Lertsanguansinchai et al. | 10.1016/j.ijcard.2022.12.023 | Aortic Stenosis |  | Mortality Prediction |  | Single |  | Tabular Clinical Data |  | Tree Based Model | 178 | Single-center | Retrospective | Mixed | Internal | K-fold cross validation + Holdout Validation | Feature Importance | None | Expert adjudication | 3 & 4 | 2023 | Yes | Yes, compared with existing standard | Yes |
| 141 | Kho et al. | 10.1007/s11357-024-01136-w | Aortic Stenosis |  | Diagnosis |  | Single |  | Wearable - Medical Grade |  | Classical Machine Learning | 149 | Multicenter | Prospective | Mixed | Internal | K-fold cross validation + Holdout Validation | None | None | Imaging/Signal Annotation Labels | 3 & 4 | 2024 | Yes | No comparison reported |  |
| 142 | Swapna et al. | 10.1140/epjp/s13360-021-01185-6 | Mitral Regurgitation |  | Diagnosis |  | Single |  | Heart Sound |  | Classical Machine Learning | 50 | Not Specified | Retrospective | Mixed | Internal | K-fold cross validation + Holdout Validation | None | Partial | Proxy Labels | 3 & 4 | 2021 | Yes | No comparison reported |  |
| 143 | Kwon et al. | 10.1161/JAHA.119.014717 | Aortic Stenosis |  | Diagnosis |  | Multimodal | Late | Multimodal | Tabular Clinical Data + Electrocardiogram | Ensemble / Hybrid Model | 25733 | Single-center | Retrospective | Mixed | Both | Holdout Validation + Independent | GRAD-CAM | None | Imaging/Signal Annotation Labels | 5 | 2020 | Yes | No comparison reported |  |
| 144 | Yang et al. | 10.1038/s41598-020-74519-6 | Aortic Stenosis |  | Diagnosis |  | Multimodal | Early | Multimodal | Seismocardiography + Gyrocardiography | Tree Based Model | 34 | Single-center | Prospective | Mixed | Internal | K-fold cross validation + Holdout Validation | None | None | Imaging/Signal Annotation Labels | 3 & 4 | 2020 | Yes | No comparison reported |  |
| 145 | Kwon et al. | 10.1016/j.jelectrocard.2020.02.008 | Mitral Regurgitation |  | Diagnosis |  | Single |  | Electrocardiogram |  | CNN Based Model | 24202 | Single-center | Retrospective | Mixed | Both | Holdout Validation + Independent | GRAD-CAM | None | Imaging/Signal Annotation Labels | 5 | 2020 | Yes | No comparison reported |  |
| 146 | Tse et al. | 10.1111/eci.13321 | Mitral Regurgitation |  | Mixed | Mortality Prediction, Complication Risks | Single |  | Tabular Clinical Data |  | Probabilistic Based Model | 706 | Single-center | Retrospective | Mixed | Internal | Holdout Validation | None | None | Diagnosis/Procedure Codes | 3 & 4 | 2020 | Yes | No comparison reported |  |
| 147 | Sankararaman | 10.1140/epjs/s11734-021-00326-3 | Aortic Stenosis |  | Diagnosis |  | Single |  | Heart Sound |  | Classical Machine Learning | 40 | Not Specified | Retrospective | Mixed | Internal | K-fold cross validation | None | None | Proxy Labels | 3 & 4 | 2021 | Yes | No comparison reported |  |
| 148 | Mejia et al. | 10.1371/journal.pone.0199277 | Mixed | As, AR, MS, MR, TS, TR | Mortality Prediction |  | Single |  | Tabular Clinical Data |  | Tree Based Model | 2919 | Single-center | Prospective | Mixed | Internal | K-fold cross validation | Feature Importance | None | Diagnosis/Procedure Codes | 3 & 4 | 2018 | Yes | Yes, compared with existing standard | Yes |
| 149 | Vijesh et al. | 10.1063/5.0102120 | Aortic Stenosis |  | Diagnosis |  | Single |  | Heart Sound |  | Classical Machine Learning | 60 | Not Specified | Retrospective | Mixed | Internal | K-fold cross validation | None | Partial | Proxy Labels | 3 & 4 | 2022 | Yes | No comparison reported |  |
| 150 | Singh et al. | 10.1109/TIM.2025.3540129 | Mixed | AS, AR, MS, MR, TR | Diagnosis |  | Single |  | Wearable - Medical Grade |  | Ensemble / Hybrid Model | 129 | Not Specified | Retrospective | Mixed | Internal | K-fold cross validation | Custom | Partial | Proxy Labels | 3 & 4 | **2025** | **No** | **No comparison reported** |  |
| 151 | Murayshid et al. | 10.1007/978-3-031-85908-3_35 | Mixed | MS, MR, AS, MVP | Diagnosis |  | Single |  | Heart Sound |  | Tree Based Model | 1000 | Not Specified | Retrospective | Mixed | Internal | Holdout Validation | None | Partial | Proxy Labels | 3 & 4 | 2025 | Yes | No comparison reported |  |
| 152 | Satyasai et al. | 10.1109/JSEN.2024.3511633 | Mixed | MS, MR, AS, MVP | Diagnosis |  | Single |  | Heart Sound |  | Ensemble / Hybrid Model | 1000 | Not Specified | Retrospective | Mixed | Internal | Holdout Validation | None | Partial | Proxy Labels | 3 & 4 | 2025 | No | No comparison reported |  |
| 153 | Kang et al. | 10.1145/3749548 | Aortic Stenosis |  | Diagnosis |  | Single |  | Wearable - Consumer Grade |  | Ensemble / Hybrid Model | 325 | Single-center | Prospective | Mixed | Internal | K-fold cross validation | GRAD-CAM | None | Expert adjudication | 3 & 4 | 2025 | No | No comparison reported |  |
| 154 | Nehary & Rajan. | 10.1109/OJIM.2025.3605226 | Mixed | MVP, MR, AS | Diagnosis |  | Single |  | Heart Sound |  | CNN Based Model | 2868 | Multicenter | Retrospective | Mixed | Internal | K-fold cross validation + Holdout Validation | None | Partial | Proxy Labels | 3 & 4 |  | No | No comparison reported |  |
| 155 | Julakanti et al. | 10.1093/ehjdh/ztae085 | Aortic Stenosis |  | Multiple | Disease Progression, Mortality Prediction | Single |  | Tabular Clinical Data |  | Tree Based Model | 9611 | Single-center | Retrospective | Mixed | Internal | K-fold cross validation + Holdout Validation | Feature Importance | None | Mixed | 5 | 2024 | No | No comparison reported |  |
| 156 | Huang et al. | 10.21037/qims-2025-120 | Mitral Regurgitation |  | Diagnosis |  | Single |  | Echocardiography |  | CNN Based Model | 266 | Single-center | Retrospective | Mixed | Internal | K-fold cross validation + Holdout Validation | GRAD-CAM | None | Expert adjudication | 3 & 4 | 2025 | Yes | No comparison reported |  |
| 157 | Gan et al. | 10.3389/fmed.2025.1587307 | Aortic Stenosis |  | Diagnosis |  | Single |  | Echocardiography |  | CNN Based Model | 599 | Single-center | Retrospective | Mixed | Both | Holdout Validation + Independent | None | Partial | Proxy Labels | 5 | 2025 | Yes | No comparison reported |  |
| 158 | Li et al. | 10.1186/s12872-025-04759-9 | Aortic Stenosis |  | Complication Risks |  | Single |  | Tabular Clinical Data |  | Tree Based Model | 987 | Single-center | Retrospective | Mixed | Internal | Holdout Validation | SHapley Additive exPlanations | None | Diagnosis/Procedure Codes | 3 & 4 | 2025 | Yes | No comparison reported |  |
| 159 | Poterucha et al. | 10.1038/s41586-025-09227-0 | Mixed | AR, AS, MR, TR, PR | Diagnosis |  | Single |  | Electrocardiogram |  | CNN Based Model | 230 318 | Multicenter | Retrospective | Mixed | Both | Holdout Validation + Independent | None | Partial | Rule-Based Phenotypes | 5 | 2025 | No | Yes, compared with cardiologist diagnosis | Yes |
| 160 | Huang et al. | 10.1109/ISBI60581.2025.10981205 | Aortic Stenosis |  | Diagnosis |  | Multimodal | Intermediate | Multimodal | Echocardiography 2d Cine + Echocardiography Doppler | Ensemble / Hybrid Model | 599 | Single-center | Retrospective | Mixed | Both | Holdout Validation + Independent | None | Partial | Proxy Labels | 5 | 2025 | Yes | No comparison reported |  |
| 161 | Wang et al. | 10.2196/70587 | Aortic Stenosis |  | Mixed | Mortality Prediction, Complication Risks | Single |  | Tabular Clinical Data |  | Classical Machine Learning | 326 | Multicenter | Retrospective | Mixed | Internal | Holdout Validation | SHapley Additive exPlanations | None | Mixed | 3 & 4 | 2025 | Yes | No comparison reported |  |
| 162 | Liang et al. | 10.1093/eurheartj/ehaf448 | Multiple | MR, AR, TR | Disease Progression |  | Single |  | Electrocardiogram |  | CNN Based Model | 128554 | Single-center | Retrospective | Mixed | Both | Holdout Validation + Independent | Custom | None | Expert adjudication | 5 | 2025 | No | No comparison reported |  |
| 163 | El Ouahidi et al. | 10.1038/s41598-024-76128-z | Aortic Stenosis |  | Complication Risks |  | Single |  | Tabular Clinical Data |  | Classical Machine Learning | 520 | Single-center | Retrospective | Mixed | Internal | K-fold cross validation | SHapley Additive exPlanations | Partial | Expert adjudication | 3 & 4 | 2024 | Yes | No comparison reported |  |
| 164 | Al-Alusi et al. | 10.1016/j.jcmg.2025.08.011 | Mitral Valve Prolapse |  | Diagnosis |  | Single |  | Echocardiography |  | CNN Based Model | 16902 | Multicenter | Retrospective | Mixed | Both | Holdout Validation + Independent | Saliency Map | None | Expert adjudication | 5 | 2025 | No | No comparison reported |  |
| 165 | Zhou et al. | 10.1016/j.jacadv.2025.101993 | Multiple | AS, AR, MS, MR | Diagnosis |  | Single |  | Heart Sound |  | Classical Machine Learning | 514 | Single-center | Ambispective | Mixed | Internal | Holdout Validation | None | None | Expert adjudication | 3 & 4 | 2025 | Yes | No comparison reported |  |
| 166 | Kho et al. | 10.1007/s11357-024-01136-w | Aortic Stenosis |  | Diagnosis |  | Single |  | Wearable - Medical Grade |  | Classical Machine Learning | 149 | Single-center | Prospective | Mixed | Internal | Holdout Validation | None | None | Expert adjudication | 3 & 4 | 2024 | Yes | No comparison reported |  |
| 167 | Tomii et al. | 10.1016/j.jacadv.2025.102168 | Aortic Stenosis |  | Complication Risks |  | Single |  | Tabular Clinical Data |  | Tree Based Model | 2937 | Single-center | Retrospective | Mixed | Internal | K-fold cross validation + Holdout Validation | SHapley Additive exPlanations | Partial | Expert adjudication | 3 & 4 | 2025 | Yes | No comparison reported |  |
| 168 | Kwiecinski et al. | 10.1016/j.pcad.2025.04.007 | Aortic Stenosis |  | Mortality Prediction |  | Single |  | Tabular Clinical Data |  | Tree Based Model | 631 | Single-center | Retrospective | Mixed | Both | K-fold cross validation + Independent | SHapley Additive exPlanations | None | External Linkage Outcome | 5 | 2025 | Yes | Yes, compared with existing standard | Yes |
| 169 | Kurmanaliyev et al. | 10.3390/medicina61030374 | Aortic Stenosis |  | Mixed | Mortality Prediction, Complication Risks | Single |  | Tabular Clinical Data |  | Tree Based Model | 224 | Single-center | Retrospective | Mixed | Internal | Holdout Validation | SHapley Additive exPlanations | None | Mixed | 3 & 4 | 2025 | No | No comparison reported |  |
| 170 | Wu et al. | 10.1093/ehjimp/qyae086 | Mitral Valve Prolapse |  | Diagnosis |  | Single |  | Echocardiography |  | CNN Based Model | 473 | Single-center | Retrospective | Mixed | Internal | Holdout Validation | None | None | Expert adjudication | 3 & 4 | 2024 | Yes | No comparison reported |  |
| 171 | Aslam et al. | 10.1038/s41598-024-67973-z | Mixed | MS, MR, AS, AR, TR, PS, TS | Diagnosis |  | Single |  | Tabular Clinical Data |  | Classical Machine Learning | 624 | Single-center | Retrospective | Mixed | Internal | K-fold cross validation | None | None | Expert adjudication | 3 & 4 | 2024 | Yes | No comparison reported |  |
| 172 | Zheng et al. | 10.3389/fcvm.2024.1410859 | Mitral Regurgitation |  | Complication Risks |  | Single |  | MRI |  | Tree Based Model | 33 | Single-center | Prospective | Mixed | Internal | Holdout Validation | SHapley Additive exPlanations | None | Registry/Trial Endpoints | 3 & 4 | 2024 | Yes | No comparison reported |  |
| 173 | Cruz et al. | 10.1016/j.surg.2024.07.011 | Mitral Regurgitation |  | Mortality Prediction |  | Single |  | Tabular Clinical Data |  | Probabilistic Based Model | 2447 | Multicenter | Retrospective | Mixed | Internal | K-fold cross validation + Holdout Validation | SHapley Additive exPlanations | None | External Linkage Outcome | 3 & 4 | 2024 | Yes | No comparison reported |  |
| 174 | Mekahlia et al. | 10.1016/j.medengphy.2025.104302 | Mixed | MS, MR, AS, MVP | Diagnosis |  | Single |  | Heart Sound |  | CNN Based Model | 1000 | Not Specified | Retrospective | Mixed | Internal | K-fold cross validation | None | Partial | Proxy Labels | 3 & 4 | 2025 | Yes | No comparison reported |  |
| 175 | Itelman et al. | 10.1016/j.jacadv.2025.102121 | Aortic Stenosis |  | Disease Progression |  | Single |  | Tabular Clinical Data |  | Tree Based Model | 3443 | Single-center | Retrospective | Mixed | Internal | Holdout Validation | SHapley Additive exPlanations | None | Mixed | 3 & 4 | 2025 | Yes | No comparison reported |  |
| 176 | Mustafa et al. | 10.1016/j.acvd.2024.08.008 | Aortic Stenosis |  | Readmission Risks |  | Single |  | Tabular Clinical Data |  | Feedforward / ANN | 25874 | Multicenter | Retrospective | Mixed | Internal | Holdout Validation | Feature Importance | None | Diagnosis/Procedure Codes | 3 & 4 | 2025 | Yes | No comparison reported |  |
| 177 | Malik et al. | 10.1016/j.jtcvs.2025.07.017 | Mitral Regurgitation |  | Complication Risks |  | Single |  | Tabular Clinical Data |  | Tree Based Model | 444 | Single-center | Retrospective | Mixed | Internal | Out-of-bag (OOB) internal cross-validation | Feature Importance | None | Expert adjudication | 3 & 4 | 2025 | Yes | No comparison reported |  |
| 178 | Barros Filho et al. | 10.3390/jimaging11080272 | Mitral Stenosis |  | Diagnosis |  | Single |  | Echocardiography |  | CNN Based Model | 30 | Single-center | Retrospective | Mixed | Internal | Holdout Validation | None | None | Expert adjudication | 3 & 4 | 2025 | No | No comparison reported |  |
| 179 | Sieciński & Grzegorzek | 10.1016/j.cmpb.2025.108925 | Mixed | AS, TR, MS, MR | Diagnosis |  | Single |  | Wearable - Medical Grade |  | Classical Machine Learning | 59 | Multicenter | Retrospective | Mixed | Internal | K-fold cross validation | None | Partial | Proxy Labels | 3 & 4 | 2025 | Yes | No comparison reported |  |
| 180 | Otomo et al. | 10.1253/circrep.CR-24-0182 | Aortic Stenosis |  | Mortality Prediction |  | Single |  | Tabular Clinical Data |  | Tree Based Model | 252 | Single-center | Retrospective | Mixed | Internal | K-fold cross validation + Holdout Validation | Mixed | None | Expert adjudication | 3 & 4 | 2025 | Yes | No comparison reported |  |
| 181 | Wu et al. | 10.1109/TMI.2025.3609319 | Aortic Stenosis |  | Diagnosis |  | Multimodal | Intermediate | Multimodal | Tabular Clinical Data + Echocardiography | Transformer Based Model | 2627 | Single-center | Retrospective | Mixed | Both | Holdout Validation + Independent | None | None | Expert adjudication | 5 | 2025 | Yes | No comparison reported |  |
| 182 | Li et al. | 10.1097/MD.0000000000044556 | Mixed | AS, AR, MS, MR, TR | Mortality Prediction |  | Single |  | Tabular Clinical Data |  | Feedforward / ANN | 443 | Single-center | Retrospective | Mixed | Internal | K-fold cross validation | Feature Importance | None | External Linkage Outcome | 3 & 4 | 2025 | No | No comparison reported |  |
| 183 | Elkouahy et al. | 10.26599/AUDT.2025.240067 | Aortic Stenosis |  | Diagnosis |  | Single |  | Echocardiography |  | CNN Based Model | 577 | Single-center | Retrospective | Mixed | Internal | Holdout Validation | None | Partial | Proxy Labels | 5 | 2025 | Yes | No comparison reported |  |
| 184 | Sakuma et al. | 10.1007/s00380-025-02546-2 | Mitral Regurgitation |  | Diagnosis |  | Single |  | Electrocardiogram |  | Ensemble / Hybrid Model | 19170 | Single-center | Retrospective | Mixed | Internal | K-fold cross validation | GRAD-CAM | None | Expert adjudication | 3 & 4 | 2025 | Yes | No comparison reported |  |
| 185 | Vairo et al. | 10.3390/jcm14165863 | Mitral Regurgitation |  | Treatment Response |  | Single |  | Tabular Clinical Data |  | Tree Based Model | 80 | Single-center | Retrospective | Mixed | Internal | K-fold cross validation | Feature Importance | None | Expert adjudication | 3 & 4 | 2025 | Yes | No comparison reported |  |
| 186 | Vrudhula et al. | doi:10.1001/jamacardio.2025.0498 | Tricuspid Regurgitation |  | Diagnosis |  | Single |  | Echocardiography |  | CNN Based Model | 47312 | Single-center | Retrospective | Mixed | Both | Holdout Validation + Independent | Saliency Map | None | Expert adjudication | 5 | 2025 | No | No comparison reported |  |
| 187 | Long et al. | 10.1093/eurheartj/ehaf248 | Multiple | AR, MR, TR | Multiple | Diagnosis, Disease Progression | Single |  | Echocardiography |  | CNN Based Model | 35711 | Multicenter | Retrospective | Mixed | Both | Holdout Validation + Independent | None | None | Expert adjudication | 5 | 2025 | No | No comparison reported |  |
| 188 | Rustamovna et al. | 10.5281/2enodo.15364876 | Mixed | AS, AR, MS, MR | Diagnosis |  | Single |  | Echocardiography |  | CNN Based Model | 500 | Multicenter | Retrospective | Mixed | Both | Holdout Validation + Independent | GRAD-CAM | None | Expert adjudication | 5 | 2025 | No | Yes, compared with cardiologist diagnosis | Yes |
| 189 | Vasileios et al. | 10.1080/14796678.2025.2498866 | Aortic Stenosis |  | Complication Risks |  | Single |  | Tabular Clinical Data |  | Tree Based Model | 469 | Single-center | Retrospective | Mixed | Internal | Holdout Validation | None | None | Rule-Based Phenotypes | 3 & 4 | 2025 | Yes | No comparison reported |  |
| 190 | Gu et al. | 10.1016/j.media.2025.103600 | Aortic Stenosis |  | Diagnosis |  | Single |  | Echocardiography |  | CNN Based Model | 2572 | Single-center | Retrospective | Mixed | Both | Holdout Validation + Independent | None | Partial | Expert adjudication | 5 | 2025 | Yes | No comparison reported |  |
| 191 | Boeckling et al. | 10.1161/JAHA.124.037296 | Aortic Stenosis |  | Mortality Prediction |  | Single |  | Tabular Clinical Data |  | Tree Based Model | 378 | Single-center | Prospective | AUROC | Internal | K-fold cross validation | None | None | External Linkage Outcome | 3 & 4 | 2025 | No | Yes, compared with existing standard | Yes |
| 192 | Hangaragi et al. | 10.1038/s41598-025-92395-w | Mixed | AS, MS, TR, PR | Diagnosis |  | Multimodal | Early | Multimodal | Electrocardiogram + Heart Sound | CNN Based Model |  | Not Specified | Retrospective | Mixed | Internal | K-fold cross validation | None | None | Proxy Labels | 3 & 4 | 2025 | Yes | No comparison reported |  |
| 193 | Zhu et al. | 10.1016/j.hjc.2024.04.003 | Mitral Regurgitation |  | Complication Risks |  | Single |  | Tabular Clinical Data |  | Classical Machine Learning | 231 | Single-center | Retrospective | Mixed | Internal | K-fold cross validation | Feature Importance | None | Expert adjudication | 3 & 4 | 2025 | Yes | No comparison reported |  |
| 194 | Sotelo et al. | 10.1016/j.ibmed.2025.100201 | Aortic Stenosis |  | Disease Progression |  | Single |  | Tabular Clinical Data |  | Ensemble / Hybrid Model | 3355 | Multicenter | Retrospective | Mixed | Both | Holdout Validation + Independent | None | None | Expert adjudication | 5 | 2025 | Yes | No comparison reported |  |
| 195 | Park et al. | 10.1016/j.ebiom.2025.105560 | Aortic Stenosis |  | Diagnosis |  | Single |  | Echocardiography |  | Ensemble / Hybrid Model | 8427 | Multicenter | Retrospective | Mixed | Both | Holdout Validation + Independent | GRAD-CAM | None | Expert adjudication | 5 | 2025 | Yes | No comparison reported |  |

Supplementary Table 2: PROBAST + AI risk of bias analysis of the in included studies

| **DOI** | **Reference name** | **Model Development - Participants** | **Model Development - Predictors** | **Model Development - Outcomes** | **Model Development - Analysis** | **Model Evaluation - Participants** | **Model Evaluation - Predictors** | **Model Evaluation- Outcomes** | **Model Evaluation - Analysis** |
| --- | --- | --- | --- | --- | --- | --- | --- | --- | --- |
| 10.1161/CIRCULATIONAHA.124.068996 | Long et al. | Low | Low | Low | Unclear | Low | Low | Low | Unclear |
| 10.1093/eurheartj/ehad871 | Hausleiter et al. | Low | Low | Low | Unclear | Low | Low | Low | Low |
| 10.1155/2023/7382316 | Ding et al. | High | Low | Unclear | Unclear | High | Low | Unclear | High |
| 10.1161/CIRCULATIONAHA.124.069047 | Vrudhula et al. | Low | Low | Low | Unclear | Low | Low | Low | Low |
| 10.1093/ehjqcco/qcad002 | Kwiecinski et al. | Low | Low | Low | High | Low | Low | Low | Unclear |
| 10.1093/ehjdh/ztad006 | Anand et al. | Low | Unclear | Low | Low | Low | Unclear | Low | Low |
| 10.1016/j.carrev.2020.06.017 | Hernandez-Suarez et al. | High | Unclear | Low | Low | High | Unclear | Low | High |
| 10.1016/j.jacadv.2024.101135 | Shimoni et al. | Low | Low | Low | Low | Low | Unclear | Low | Low |
| 10.1016/j.athoracsur.2020.05.107 | Kilic et al. | Unclear | Low | Low | Low | Unclear | Low | Low | Low |
| 10.1109/EMBC44109.2020.9175151 | Hata et al. | Low | Low | Low | High | Low | Low | Low | Unclear |
| 10.1016/j.compbiomed.2023.106734 | Jamil et al. | High | Low | Low | Low | High | Low | Low | Unclear |
| 10.1016/j.ejrad.2023.111150 | Theiset al. | Low | Low | Low | Unclear | Low | Low | Low | Unclear |
| 10.1016/j.cmpb.2021.105940 | Alkhodari & Fraiwan | High | Low | Low | Unclear | High | High | Low | Unclear |
| 10.1016/j.jcmg.2021.08.015 | Yang et al. | Low | Low | Low | Low | Low | Low | Low | Low |
| 10.1016/j.carrev.2020.08.010 | Agasthi et al. | Unclear | Low | Low | High | Unclear | Low | Low | Low |
| 10.1111/jocs.16060 | Kachroo et al. | Unclear | Low | Unclear | High | Unclear | Low | Unclear | Unclear |
| 10.1038/s43856-023-00240-w | Vaid et al. | Low | Low | Low | Low | Low | Low | Low | Low |
| 10.1161/JAHA.121.024168 | Cheng et al. | Low | Low | Low | Low | Low | Low | Low | Low |
| 10.1016/j.surg.2024.07.011 | Cruz et al | High | Low | Low | Unclear | High | Low | Low | Low |
| 10.1007/s00392-020-01691-0 | Gomes et al. | Unclear | Unclear | Low | Unclear | Unclear | Unclear | Low | Unclear |
| 10.1016/j.jcin.2021.03.024 | Navarese et al. | Low | Low | Low | Low | Low | Low | Low | Low |
| 10.1038/s41598-021-82403-0 | Castela Forte et al. | Low | Low | Low | Low | Low | Low | Low | Low |
| 10.3390/bioengineering10111319 | Huang et al. | High | Low | Unclear | High | High | Low | Unclear | Unclear |
| 10.3390/ bioengineering8090117 | Penso et al. | Unclear | Low | Low | Unclear | Unclear | Low | Low | Low |
| 10.1007/s11517-023-02827-w | Roy et al | High | Unclear | High | High | High | Unclear | High | High |
| 10.1016/j.hjc.2024.04.003 | Zhu et al. | Unclear | Low | Unclear | Unclear | Unclear | Low | Unclear | Unclear |
| 10.1016/j.jacadv.2024.101234 | Sanabria et al. | Low | Low | Unclear | Unclear | Low | Low | Unclear | Low |
| 10.1016/j.cmpb.2023.107906 | Ma et al. | High | Low | Unclear | High | High | Unclear | Low | High |
| 10.1136/ openhrt-2023-002540 | Zisiopoulou et al. | Unclear | Low | Low | Unclear | Unclear | Low | Low | Unclear |
| 10.1016/j.jacadv.2023.100446 | Tison et al. | Low | Low | Low | Unclear | Low | Low | Low | Unclear |
| 10.1016/j.xjtc.2023.11.011 | Dasi et al. | Unclear | Unclear | Low | Low | Unclear | Unclear | Low | Low |
| 10.1088/1361-6560/ad22a4 | Kim et al. | High | Low | Low | High | High | Low | Low | Unclear |
| 10.3390/jcm13133691 | Barbieri et al. | Low | Low | Low | Unclear | Low | Low | Low | Unclear |
| 10.14744/SEMB.2024.00836 | Erdogan et al | Unclear | Low | Unclear | High | Unclear | Low | Unclear | Unclear |
| 10.3389/fcvm.2021.787246 | Lopes et al. | Low | Low | Low | Unclear | Low | Low | Low | High |
| 10.3390/bioengineering8020022 | Mamprin et al. | Low | Low | Low | Unclear | Low | Low | Low | Low |
| 10.3390/bioengineering11010058 | Ribeiro et al. | High | High | Unclear | High | High | High | Unclear | High |
| 10.1016/j.heliyon.2023.e17139 | Lopes et al. | Low | Unclear | Unclear | Low | Low | Unclear | Unclear | Unclear |
| 10.3390/jcdd10020087 | Zhu et al. | Unclear | Low | Low | Unclear | Unclear | Low | Low | Unclear |
| 10.1063/1.4926642 | Suboh et al. | High | High | High | Unclear | High | High | High | High |
| 10.1109/INDICON56171.2022.10039701 | Das et al. | High | Low | Low | Unclear | High | Low | Low | Unclear |
| 10.1109/ISOCC59558.2023.10396555 | Chang et al. | High | Unclear | Unclear | Unclear | High | Unclear | Unclear | High |
| 10.1080/23311916.2020.1856757 | Joukhadar et al. | High | Unclear | Unclear | High | High | Unclear | High | High |
| 10.3390/app12083780 | Flores-Alonso et al. | High | Low | Low | Unclear | High | Low | Low | Low |
| 10.1002/cpe.7232 | Yildirim | High | Low | Low | Unclear | High | Low | Low | Unclear |
| 10.1016/j.bspc.2023.104805 | Maity et al. | High | Low | Low | Unclear | High | Low | Low | Unclear |
| 10.1016/j.cmpb.2020.105604 | Oh et al. | High | Low | Low | Unclear | High | Low | Low | Unclear |
| 10.1109/TIM.2022.3163156 | Karhade et al. | High | Low | Low | Unclear | High | Low | Low | Unclear |
| 10.23919/CinC53138.2021.9662695 | Elnaggar et al. | High | Low | Unclear | Unclear | High | Low | Unclear | Unclear |
| 10.1016/j.eswa.2023.121772 | Tartarisco et al. | High | Low | Low | High | High | Low | Low | High |
| 10.1016/j.bspc.2023.105086 | Roy et al. | High | Low | Unclear | High | High | Low | Unclear | High |
| 10.1109/TIM.2023.3274174 | Bhardwaj et al. | High | Low | Low | Unclear | High | Low | Low | Unclear |
| 10.1109/JSEN.2022.3196263 | Chowdhury et al. | High | Low | Low | Unclear | High | Low | Unclear | Unclear |
| 10.1002/ima.22885 | Vafaeezadeh et al. | Low | Low | Low | Unclear | Low | Low | Low | Unclear |
| 10.1016/j.ins.2021.01.088 | Tuncer et al. | High | Low | Low | Unclear | High | Low | Low | Unclear |
| 10.4103/jicc.jicc_44_23 | Mani et al. | Low | Low | Low | Unclear | Low | Low | Low | Low |
| 10.1504/IJMEI.2021.115970 | Hafiz et al. | High | Unclear | Unclear | High | High | Unclear | Unclear | High |
| 10.2147/JIR.S453100 | Yu et al. | High | Unclear | Unclear | High | High | Unclear | Unclear | High |
| 10.18632/aging.205835 | Lin et al | Low | Low | Low | Low | Low | Low | Low | Low |
| 10.1038/s41598-024-63022-x | Bruggermann et al. | Low | Low | Low | Low | Low | Low | Low | Low |
| 10.1038/s41746-024-01130-8 | Kalmady et al | Low | Low | Unclear | Low | Low | Low | Unclear | Low |
| 10.1109/ACCESS.2024.3451660 | Al-Tam et al. | High | Low | Low | Unclear | High | Low | Low | Unclear |
| 10.1109/TBME.2019.2942741 | Yang et al. | High | Low | Low | High | High | Low | Unclear | High |
| 10.1109/ACCESS.2024.3357946 | Jumphoo et al. | High | Low | Low | High | High | Low | Low | Unclear |
| 10.1109/JSEN.2023.3289109 | Morshed et al. | High | Low | Low | Unclear | High | Low | Low | Unclear |
| 10.3389/fcvm.2023.1170804 | Waaler et al. | Low | Low | Unclear | Low | Low | Low | Unclear | Low |
| 10.23919/CCC55666.2022.9901903 | Su et al. | High | Low | Unclear | Unclear | High | Low | Unclear | Unclear |
| 10.1016/j.bspc.2021.103445 | Khan et al. | High | Low | Low | Unclear | High | Low | Low | Unclear |
| 10.1016/j.amjmed.2022.04.032 | Ghanayim et al | Unclear | Unclear | High | Unclear | Unclear | High | Unclear | Unclear |
| 10.1016/j.ijcha.2024.101368 | Jiang et al. | Low | Low | Low | Unclear | Low | Low | Low | Unclear |
| 10.1093/jigpal/jzv009 | Hassanien et al. | High | Low | High | High | High | Low | High | High |
| 10.3390/s23249834 | Shiraga et al. | Unclear | Low | Low | High | Unclear | Low | Low | Unclear |
| 10.1038/s41598-022-18293-7 | Al-Issa & Alqudah | High | Low | Low | Unclear | High | Low | Low | Unclear |
| 10.1007/s10462-022-10184-7 | Zeng et al. | High | Low | Low | Unclear | High | Low | Low | Unclear |
| 10.1016/j.jacc.2022.05.029 | Elias et al. | Unclear | Low | Low | Low | Unclear | Low | Low | Low |
| 10.1136/bmjopen-2022-067977 | Gaye et al. | Unclear | Low | Low | Unclear | Unclear | Low | Low | Unclear |
| 10.1016/j.cmpb.2024.108037 | Avola et al. | Low | Low | Low | High | Low | Low | Low | High |
| 10.1002/adts.202300549 | Li et al. | High | Low | Low | Unclear | High | Low | Low | Unclear |
| 10.3390/jcdd8040044 | Penso et al. | Low | Low | Low | Unclear | Low | Low | Low | Unclear |
| 10.1007/s00034-023-02588-9 | Zhang et al. | High | Low | Low | Unclear | High | Low | Low | Unclear |
| 10.1016/j.bspc.2023.105272 | Shuvo et al. | High | Low | Low | Unclear | High | Low | Low | Unclear |
| 10.1007/978-3-031-43990-2_23 | Xiao et al. | Unclear | Low | Low | Unclear | Unclear | Low | Low | Unclear |
| 10.1109/ICUMT48472.2019.8970870 | Dubey et al. | High | Low | Low | Unclear | High | Low | Low | Unclear |
| 10.1109/OJIM.2023.3320765 | Roy et al. | High | Unclear | Unclear | Unclear | High | Unclear | Unclear | Unclear |
| 10.1016/j.bspc.2023.105265 | Qi et al. | Low | Low | Low | Low | Low | Low | Low | Low |
| 10.1007/s10462-021-09969-z | Zeng et al. | High | Low | Low | Unclear | High | Low | Low | Unclear |
| 10.1093/icvts/ivad176 | Kang et al. | Low | Low | Unclear | Unclear | Low | Low | Unclear | Unclear |
| 10.1136/openhrt-2023-002417 | Deb et al. | Low | Low | Low | Low | Low | Low | Low | Low |
| 10.1016/j.cpcardiol.2023.102143 | Zahid et al. | Low | Low | Low | Unclear | Low | Low | Low | Unclear |
| 10.1093/eurheartj/ehad456 | Holste et al. | Low | Low | Low | Low | Low | Low | Low | Low |
| 10.1007/s10439-023-03342-7 | Ebrahimkhani et al. | Low | Unclear | Low | Unclear | Low | Unclear | Low | Unclear |
| 10.1007/s00034-022-02124-1 | Nguyen et al. | High | Low | Low | High | High | Low | Low | High |
| 10.3389/fcvm.2023.1153814 | Namasivayam et al. | Low | Unclear | Unclear | Unclear | Low | Unclear | Unclear | Unclear |
| 10.1016/j.jksuci.2021.12.019 | Arslan & Karhan | High | Low | Low | Unclear | High | Low | Low | Unclear |
| 10.1109/TIM.2023.3240995 | Rajeshwari et al. | High | Low | Low | High | High | Low | Low | High |
| 10.3390/electronics10040495 | Wahlang et al. | Low | Low | Low | Unclear | Low | Low | Low | Unclear |
| 10.1016/j.jcmg.2022.12.008 | Sánchez-Puente et al. | Low | Low | Low | Low | Low | Low | Low | Low |
| 10.1016/ S2589-7500(23)00107-3 | Ueda et al. | Low | Low | Low | Low | Low | Low | Low | Low |
| 10.3389/fcvm.2022.866257 | Zhou et al. | Low | Low | Low | Unclear | Low | Low | Low | Unclear |
| 10.1007/s10489-023-04877-x | Luosang et al. | Low | Low | Low | Unclear | Low | Low | Low | Unclear |
| 10.1016/j.eswa.2023.119720 | Prabhakar & Won | High | Unclear | Unclear | Unclear | High | Unclear | Unclear | Unclear |
| 10.1093/ehjci/jead077 | Kwak et al. | Low | Low | Low | Unclear | Low | Low | Low | Unclear |
| 10.4103/ACCJ.ACCJ_13_22 | Chen et al. | Low | Low | Low | Unclear | Low | Low | Low | Unclear |
| 10.1136/openhrt-2023-002265 | Strange et al. | Low | Low | Low | Low | Low | Low | Low | Low |
| 10.1080/23311916.2018.1502906 | Kumar et al. | High | High | High | High | High | High | High | High |
| 10.1007/978-3-030-17971-7_23 | Mustafić et al. | High | High | High | High | High | High | High | High |
| 10.3390/electronics12234835 | Elvas et al. | Unclear | Low | Low | High | Unclear | Low | Low | Unclear |
| 10.1109/INDICON56171.2022.10039770 | Rishal & Satija | High | Low | Low | Unclear | High | Low | Low | Unclear |
| 10.1016/j.cpcardiol.2022.101464 | Zhou et al. | Low | Low | Low | Unclear | Low | Low | Low | Unclear |
| 10.1093/ehjci/jead009 | Heitzinger et al. | Low | Low | Low | Low | Low | Low | Low | Low |
| 10.1038/s41598-023-37358-9 | Alhwiti et al. | Unclear | Low | Low | Low | Unclear | Low | Low | Low |
| 10.3389/fcvm.2023.1112797 | Zheng et al. | Low | Low | Unclear | Unclear | Low | Low | Unclear | Unclear |
| 10.1016/j.echo.2023.01.006 | Wessler et al. | Low | Low | Low | Low | Low | Low | Low | Low |
| 10.3389/fcvm.2023.1130152 | Asheghan et al. | Low | Low | Low | Unclear | Low | Low | Low | Unclear |
| 10.1016/j.bea.2022.100035 | Roy et al. | High | Low | Low | Unclear | High | Low | Low | Unclear |
| 10.1016/j.compbiomed.2022.105599 | Barua et al. | Unclear | Low | Low | Unclear | Unclear | Low | Low | Unclear |
| 10.1111/exsy.13411 | Talal et al. | High | Low | Low | Unclear | High | Low | Low | Unclear |
| 10.1093/ehjdh/ztac029 | Makimoto et al. | Unclear | Low | Low | Unclear | Unclear | Low | Low | Unclear |
| 10.1109/ICCECE51049.2023.10085513 | Roy et al. | High | Low | Unclear | Unclear | High | Low | Unclear | Unclear |
| 10.1016/j.carrev.2022.07.024 | Bansal et al. | Low | Low | Low | Low | Low | Low | Low | Low |
| 10. 1136/openhrt-2022-001990 | Namasivayam et al. | Low | Low | Low | Unclear | Low | Low | Low | Unclear |
| 10.1016/j.bspc.2022.103929 | Arslan | High | Low | Low | Unclear | High | Low | Low | Unclear |
| 10.1002/clc.23826 | Voigt et al. | Unclear | Low | Low | Unclear | Unclear | Low | Low | Unclear |
| 10.1038/s41598-024-61685-0 | Mohammadyari et al. | Low | Low | Low | Unclear | Low | Low | Low | Unclear |
| 10.1148/ryai.210221 | Ueda et al. | Unclear | Low | Low | Unclear | Unclear | Low | Low | Unclear |
| 10.3390/jcdd9030086 | Wang et al. | High | Low | Unclear | Unclear | High | Low | Unclear | Unclear |
| 10.1016/j.jjcc.2021.08.029 | Sawano et al. | Low | Low | Low | Unclear | Low | Low | Low | Unclear |
| 10.1109/INDICON.2016.7839002 | Suhas et al. | High | Unclear | Unclear | High | High | Unclear | Unclear | High |
| 10.1007/978-3-031-16431-6_54 | Vimalesvaran et l. | Low | Low | Low | Unclear | Low | Low | Low | Unclear |
| 10.1109/ACCESS.2019.2916762 | Zhang et al. | Unclear | Low | Low | Unclear | Unclear | Low | Low | Unclear |
| 10.1016/j.jcin.2021.06.039 | Zweck et al. | Low | Low | Low | Low | Low | Low | Low | Low |
| 10.1007/978-3-030-87583-1_20 | Ginsberg et al. | Low | Low | Low | Unclear | Low | Low | Low | Unclear |
| 10.1038/s41598-021-03441-2 | Shokouhmand et al. | Low | Low | High | Unclear | Low | Low | High | Unclear |
| 10.1093/eurheartj/ehab153 | Cohen-Shelly et al. | Low | Low | Low | Low | Low | Low | Low | Low |
| 10.3348/kjr.2020.0099 | Kang et al. | Unclear | Low | Low | Unclear | Unclear | Low | Low | Unclear |
| 10.1111/pace.14163 | Truong et al. | Low | Low | Low | Unclear | Low | Low | Low | Unclear |
| 10.1016/j.compbiomed.2016.03.026 | Moghaddasi & Nourian | Unclear | Low | Low | Unclear | Unclear | Low | Low | Unclear |
| 10.1109/ACCESS.2021.3063129 | Shuvo et al. | High | Low | Low | Unclear | High | Low | Low | Unclear |
| 10.1007/s12471-019-1285-7 | Lopes et al. | Unclear | Low | Low | Unclear | Unclear | Low | Low | Unclear |
| 10.1016/j.ijcard.2022.12.023 | Lertsanguansinchai et al. | Low | Low | Low | Unclear | Low | Low | Low | Unclear |
| 10.1007/s11357-024-01136-w | Kho et al. | Low | Low | Low | High | Low | Low | Low | Unclear |
| 10.1140/epjp/s13360-021-01185-6 | Swapna et al. | High | Unclear | Low | Unclear | High | Unclear | Low | Unclear |
| 10.1161/JAHA.119.014717 | Kwon et al. | Low | Low | Low | Low | Low | Low | Low | Low |
| 10.1038/s41598-020-74519-6 | Yang et al. | Unclear | Low | Low | Unclear | Unclear | Low | Low | Unclear |
| 10.1016/j.jelectrocard.2020.02.008 | Kwon et al. | Low | Low | Low | Low | Low | Low | Low | Low |
| 10.1111/eci.13321 | Tse et al. | Unclear | Low | Unclear | Unclear | Unclear | Low | Unclear | Unclear |
| 10.1140/epjs/s11734-021-00326-3 | Sankararaman | High | Low | Unclear | High | High | Low | Unclear | High |
| 10.1371/journal.pone.0199277 | Mejia et al. | Low | Low | Low | Unclear | Low | Low | Low | Unclear |
| 10.1063/5.0102120 | Vijesh et al. | High | Low | Low | High | High | Low | Unclear | High |
| 10.1109/TIM.2025.3540129 | Singh et al. | High | High | Low | Low | Unclear | Unclear | High | High |
| 10.1007/978-3-031-85908-3_35 | Murayshid et al. | High | High | Low | Low | Low | Low | High | High |
| 10.1109/JSEN.2024.3511633 | Satyasai et al. | High | High | Low | Low | Low | Low | Unclear | Unclear |
| 10.1145/3749548 | Kang et al. | Low | Low | Low | Low | Low | Low | Unclear | Unclear |
| 10.1109/OJIM.2025.3605226 | Nehary & Rajan. | High | High | High | High | Unclear | Unclear | Unclear | Unclear |
| 10.1093/ehjdh/ztae085 | Julakanti et al. | Unclear | Unclear | Low | Low | Unclear | Unclear | Unclear | Unclear |
| 10.21037/qims-2025-120 | Huang et al. | Low | Low | Low | Low | Low | Low | Unclear | Unclear |
| 10.3389/fmed.2025.1587307 | Gan et al. | Unclear | Unclear | Low | Low | Unclear | Unclear | Unclear | Unclear |
| 10.1186/s12872-025-04759-9 | Li et al. | Low | Low | Low | Low | Low | Low | High | High |
| 10.1038/s41586-025-09227-0 | Poterucha et al. | Low | Low | Low | Low | Low | Low | Low | Low |
| 10.1109/ISBI60581.2025.10981205 | Huang et al. | Unclear | Unclear | Low | Low | Unclear | Unclear | Unclear | Unclear |
| 10.2196/70587 | Wang et al. | Low | Low | Low | Low | Low | Low | Unclear | Unclear |
| 10.1093/eurheartj/ehaf448 | Liang et al. | Low | Low | Low | Low | Low | Low | Low | Low |
| 10.1038/s41598-024-76128-z | El Ouahidi et al. | Low | Low | Low | Low | Low | Low | Unclear | Unclear |
| 10.1016/j.jcmg.2025.08.011 | Al-Alusi et al. | Low | Low | Low | Low | Low | Low | Low | Low |
| 10.1016/j.jacadv.2025.101993 | Zhou et al. | Low | Low | Low | Low | Low | Low | Unclear | Unclear |
| 10.1007/s11357-024-01136-w | Kho et al. | High | High | Low | Low | High | High | Unclear | Unclear |
| 10.1016/j.jacadv.2025.102168 | Tomii et al. | Low | Low | Low | Low | Low | Low | Low | Low |
| 10.1016/j.pcad.2025.04.007 | Kwiecinski et al. | Low | Low | Low | Low | Low | Low | Unclear | Unclear |
| 10.3390/medicina61030374 | Kurmanaliyev et al. | Low | Low | Low | Low | Low | Low | Unclear | Unclear |
| 10.1093/ehjimp/qyae086 | Wu et al. | Low | Low | Low | Low | Low | Low | Unclear | Unclear |
| 10.1038/s41598-024-67973-z | Aslam et al. | Low | Low | Low | Low | Low | Low | Unclear | Unclear |
| 10.3389/fcvm.2024.1410859 | Zheng et al. | Low | Low | Low | Low | Low | Low | High | High |
| 10.1016/j.surg.2024.07.011 | Cruz et al. | Low | Low | Low | Low | Low | Low | Low | Low |
| 10.1016/j.medengphy.2025.104302 | Mekahlia et al. | High | High | Low | Low | Low | Low | Unclear | Unclear |
| 10.1016/j.jacadv.2025.102121 | Itelman et al. | Low | Low | Low | Low | Low | Low | Low | Low |
| 10.1016/j.acvd.2024.08.008 | Mustafa et al. | Unclear | Unclear | Low | Low | Low | Low | Low | Low |
| 10.1016/j.jtcvs.2025.07.017 | Malik et al. | Low | Low | Low | Low | Low | Low | Unclear | Unclear |
| 10.3390/jimaging11080272 | Barros Filho et al. | Unclear | Unclear | Low | Low | Low | Low | High | High |
| 10.1016/j.cmpb.2025.108925 | Sieciński & Grzegorzek | High | High | Low | Low | Unclear | Unclear | Unclear | Unclear |
| 10.1253/circrep.CR-24-0182 | Otomo et al. | Low | Low | Low | Low | Low | Low | Unclear | Unclear |
| 10.1109/TMI.2025.3609319 | Wu et al. | Low | Low | Low | Low | Low | Low | Unclear | Low |
| 10.1097/MD.0000000000044556 | Li et al. | Unclear | Unclear | Low | Low | Low | Low | High | Unclear |
| 10.26599/AUDT.2025.240067 | Elkouahy et al. | Low | Low | Low | Low | Low | Low | Unclear | Unclear |
| 10.1007/s00380-025-02546-2 | Sakuma et al. | Low | Low | Low | Low | Low | Low | High | High |
| 10.3390/jcm14165863 | Vairo et al. | Low | Low | Low | Low | Low | Low | Unclear | Unclear |
| doi:10.1001/jamacardio.2025.0498 | Vrudhula et al. | Low | Low | Low | Low | Low | Low | Low | Low |
| 10.1093/eurheartj/ehaf248 | Long et al. | Low | Low | Low | Low | Low | Low | Low | Low |
| 10.5281/2enodo.15364876 | Rustamovna et al. | Low | Low | Low | Low | Low | Low | Unclear | Unclear |
| 10.1080/14796678.2025.2498866 | Vasileios et al. | Low | Low | Low | Low | Low | Low | High | High |
| 10.1016/j.media.2025.103600 | Gu et al. | Low | Low | Low | Low | Low | Low | Low | Low |
| 10.1161/JAHA.124.037296 | Boeckling et al. | Low | Low | Low | Low | Low | Low | High | High |
| 10.1038/s41598-025-92395-w | Hangaragi et al. | High | High | High | High | Unclear | Unclear | High | High |
| 10.1016/j.hjc.2024.04.003 | Zhu et al. | Low | Low | Low | Low | Low | Low | Unclear | Unclear |
| 10.1016/j.ibmed.2025.100201 | 10.1016/j.ibmed.2025.100201 | Low | Low | Low | Low | Low | Low | Unclear | Unclear |
| 10.1016/j.ebiom.2025.105560 | Park et al. | Low | Low | Low | Low | Low | Low | Low | Low |

Supplementary Table 3: Comparison of unimodal and multimodal models

| **Paper** | **DOI** | **reference** | **model_task** | **compared_to_unimodal** | **unimodal_performance** | **multimodal performance** | **metric** |
| --- | --- | --- | --- | --- | --- | --- | --- |
| Multi-center retrospective cohort study applying deep learning to electrocardiograms to identify left heart valvular dysfunction | 10.1038/s43856-023-00240-w | Vaid et al. | AS Diagnosis | Yes | 0,78 | 0,89 | AUROC |
|  |  | Vaid et al. | MR Diagnosis | Yes | 0,67 | 0,89 | AUROC |
| Detecting Aortic Stenosis Using Seismocardiography and Gyrocardiography combined with Convuolutional Neural Networks | 10.23919/CinC53138.2021.9662695 | Elnaggar et al. | AS Diagnosis | No |  |  |  |
| Predicting mortality after transcatheter aortic valve replacement using preprocedural CT | 10.1038/s41598-024-63022-x | Bruggermann et al. | Mortality Prediction | Yes | 0,723 | 0,725 | AUROC |
| Classification of Aortic Stenosis Using TimeFrequency Features from Chest Cardio-mechanical Signals | 10.1109/TBME.2019.2942741 | Yang et al. | AS Diagnosis | Yes | 0,974 | 0,9896 | Accuracy |
| Algorithm for predicting valvular heart disease from heart sounds in an unselected cohort | 10.3389/fcvm.2023.1170804 | Waaler et al. | AR Diagnosis | Yes | 0,634 | 0,747 | AUROC |
|  |  | Waaler et al. | AS Diagnosis | Yes | 0,979 | 0,982 | AUROC |
|  |  | Waaler et al. | MR Diagnosis | Yes | 0,558 | 0,672 | AUROC |
|  |  | Waaler et al. | MS Diagnosis | Yes | 0,922 | 0,938 | AUROC |
| Improving Valvular Pathologies and Ventricular Dysfunction Diagnostic Efficiency Using Combined Auscultation and Electrocardiography Data: A Multimodal AI Approach | 10.3390/s23249834 | Shiraga et al. | AS Diagnosis | Yes | 0,974 | 0,974 | AUROC |
|  |  | Shiraga et al. | MR Diagnosis | Yes | 0,725 | 0,781 | AUROC |
| Residual neural networks based on empirical mode decomposition for mitral regurgitation prediction | 10.1016/j.bspc.2023.105265 | Qi et al. | MR Diagnosis | No |  |  |  |
| A Deep Learning Approach to Using Wearable Seismocardiography (SCG) for Diagnosing Aortic Valve Stenosis and Predicting Aortic Hemodynamics Obtained by 4D Flow MRI | 10.1007/s10439-023-03342-7 | Ebrahimkhani et al. | AS Diagnosis | No |  |  |  |
| MemGCN: memory-augmented graph neural network for predict conduction disturbance after transcatheter aortic valve replacement | 10.1007/s10489-023-04877-x | Luosang et al. | CR Prediction | Yes | 0,683 | 0,778 | Accuracy |
| Deep learning model to detect significant aortic regurgitation using electrocardiography | 10.1016/j.jjcc.2021.08.029 | Sawano et al. | AR Diagnosis | Yes | 0,734 | 0,802 | AUROC |
| Efficient detection of aortic stenosis using morphological characteristics of cardiomechanical signals and heart rate variability parameters | 10.1038/s41598-021-03441-2 | Shokouhmand et al. | AS Diagnosis | No |  |  |  |
| Deep learning-based algorithm for detecting aortic stenosis using electrocardiography | 10.1161/JAHA.119.014717 | Kwon et al. | AS Diagnosis | Yes | 0,825 | 0,884 | AUROC |
| Classification of aortic stenosis using conventional machine learning and deep learning methods based on multi-dimensional cardio-mechanical signals | 10.1038/s41598-020-74519-6 | Yang et al. | AS Diagnosis | Yes | 0,86 | 0,93 | Accuracy |
| Semi-Supervised Multimodal Multi-Instance Learning for Aortic Stenosis Diagnosis | 10.1109/ISBI60581.2025.10981205 | Huang et al | AS Diagnosis | Yes | 0.797 | 0.835 | Accuracy |
| MultiASNet: Multimodal Label Noise Robust Framework for the Classification of Aortic Stenosis in Echocardiography | 10.1109/TMI.2025.3609319 | Wu et al. | AS Diagnosis | Yes | 0.769 | 0.804 | Accuracy |
| Integrated fusion approach for multi-class heart disease classification through ECG and PCG signals with deep hybrid neural networks | 10.1038/s41598-025-92395-w | Hangaragi et al. | Mixed Diagnosis | No |  |  |  |

Supplementary Table 4: Description of the sources of outcome labels

| Source | Description |
| --- | --- |
| Expert adjudication | Outcomes labelled by cardiologists or a blinded committee using a prespecified clinical criterion. |
| Registry/ trial endpoints | Labels imported from a disease registry or clinical trial with a centralized endpoint adjudication. |
| Diagnosis/procedure codes | Diagnosis/procedure codes like ICD 10, SNOMED, DRG for clinical and administrative outcomes. |
| NLP derived labels | Labels extracted from notes or reports via ML/NLP |
| Imaging/ signal annotation labels | Human, device or lab annotations on images/signals |
| External linkage outcomes | Labels obtained by linking to death registers, national hospitalization/claims or external sources of mortality data. |
| Proxy labels | Labels constructed from multiple sources/ external databases without standardized adjudication or databases not originally collected for the current study. |
| Patient reported outcomes | Self-reported clinical outcomes like disease status, quality of life and symptoms. |
| Wearable-derived event labels |  |

Supplementary Table 5: Definition of Terms

| **Box 1**. Definition of terms |
| --- |
| **Unimodal machine learning**  Machine learning models that learn from single, homogeneous types of data to make predictions. This includes unimodal models that use multisource learning, where data from different sources is first combined, harmonized, or otherwise transformed into a single unified representation before training begins. Once fused, the model treats the input as a single modality.  **Multimodal machine learning**  Machine learning models that jointly process or integrate two or more distinct data types, such as imaging, electrocardiograms, and structured clinical data, to learn richer representations and make predictions. In multimodal approaches, each data modality can be represented, encoded, and learned either separately or jointly, and the model explicitly combines these heterogeneous sources during training.  **Types of data fusion**   - Early fusion (feature level fusion): Involves combining features from different data sources into a single representation before feeding the model. - Late fusion (decision-level fusion): Involves using training different models per data source and combining their predictions. - Intermediate fusion (joint level): Integrates elements of both early and late fusion in a stepwise manner.   **Classical Machine Learning**  Traditional machine learning algorithms like logistic regression, support vector machines, k-nearest neighbors, which usually require manual feature extraction.  **Tree-based models**  Non-parametric models like decision trees, random forests, and gradient boosting machines, that use hierarchical tree structures. |

Supplementary Methods

Supplementary Table 6: Search Strategy

| Pubmed | ("Artificial Intelligence"[Mesh] OR "Machine Learning"[Mesh] OR "Deep Learning"[Mesh] OR "Neural Networks, Computer"[Mesh] OR "Artificial Intelligence"[TW] OR "Machine Learning"[TW] OR "Deep Learning"[TW] OR "Neural Network*"[TW])  AND  ("Heart Valve Diseases"[Mesh] OR "Heart Valve Disease*"[TW] OR "Valvular Disease*"[TW] OR "Valvular Heart Disease*"[TW] OR "Valve Disease*"[TW] OR "Valve Disorder*"[TW] OR "Valvular Disorder*"[TW] OR "Cardiac Valve Disease*"[TW] OR "Cardiac Valvular Disease*"[TW] OR "Aortic Valve Stenosis"[Mesh] OR "Aortic Stenosis"[TW] OR "Aortic Valve Insufficiency"[Mesh] OR "Aortic Insufficiency"[TW] OR "Aortic Regurgitation"[TW] OR "Mitral Valve Stenosis"[Mesh] OR "Mitral Stenosis"[TW] OR "Mitral Valve Insufficiency"[Mesh] OR "Mitral Insufficiency"[TW] OR "Mitral Regurgitation"[TW] OR "Pulmonary Valve Stenosis"[Mesh] OR "Pulmonary Stenosis"[TW] OR "Pulmonary Valve Insufficiency"[Mesh] OR "Pulmonary Insufficiency"[TW] OR "Pulmonary Regurgitation"[TW] OR "Tricuspid Valve Stenosis"[Mesh] OR "Tricuspid Stenosis"[TW] OR "Tricuspid Valve Insufficiency"[Mesh] OR "Tricuspid Insufficiency"[TW] OR "Tricuspid Regurgitation"[TW])  AND  ("Algorithms"[Mesh] OR "Algorithm"[TW] OR "Model*"[TW] OR "Method*"[TW] OR "Technique*"[TW] OR "Solution*"[TW] OR "System*"[TW]) |
| --- | --- |
| Web of Science | TS=("Artificial Intelligence" OR "Machine Learning" OR "Deep Learning" OR "Neural Network*")  AND  TS=("Heart Valve Diseases" OR "Heart Valve Disease*" OR "Valvular Disease*" OR "Valvular Heart Disease*" OR "Valve Disease*" OR "Valve Disorder*" OR "Valvular Disorder*" OR "Cardiac Valve Disease*" OR "Cardiac Valvular Disease*" OR "Aortic Valve Stenosis" OR "Aortic Stenosis" OR "Aortic Valve Insufficiency" OR "Aortic Insufficiency" OR "Aortic Regurgitation" OR "Mitral Valve Stenosis" OR "Mitral Stenosis" OR "Mitral Valve Insufficiency" OR "Mitral Insufficiency" OR "Mitral Regurgitation" OR "Pulmonary Valve Stenosis" OR "Pulmonary Stenosis" OR "Pulmonary Valve Insufficiency" OR "Pulmonary Insufficiency" OR "Pulmonary Regurgitation" OR "Tricuspid Valve Stenosis" OR "Tricuspid Stenosis" OR "Tricuspid Valve Insufficiency" OR "Tricuspid Insufficiency" OR "Tricuspid Regurgitation")  AND  TS=("Algorithms" OR "Algorithm" OR "Model*" OR "Method*" OR "Technique*" OR "Solution*" OR "System*") |
| Embase | ('artificial intelligence'.ti,ab OR 'machine learning'.ti,ab OR 'deep learning'.ti,ab OR 'neural network*'.ti,ab)  AND  ('heart valve disease*'.ti,ab OR 'valvular disease*'.ti,ab OR 'valvular heart disease*'.ti,ab OR 'valve disease*'.ti,ab OR 'valve disorder*'.ti,ab OR 'valvular disorder*'.ti,ab OR 'cardiac valve disease*'.ti,ab OR 'cardiac valvular disease*'.ti,ab OR 'aortic stenosis'.ti,ab OR 'aortic insufficiency'.ti,ab OR 'aortic regurgitation'.ti,ab OR 'mitral stenosis'.ti,ab OR 'mitral insufficiency'.ti,ab OR 'mitral regurgitation'.ti,ab OR 'pulmonary stenosis'.ti,ab OR 'pulmonary insufficiency'.ti,ab OR 'pulmonary regurgitation'.ti,ab OR 'tricuspid stenosis'.ti,ab OR 'tricuspid insufficiency'.ti,ab OR 'tricuspid regurgitation'.ti,ab)  AND  ('algorithm'.ti,ab OR 'model*'.ti,ab OR 'method*'.ti,ab OR 'technique*'.ti,ab OR 'solution*'.ti,ab OR 'system*'.ti,ab) |

*Eligibility criteria*

Our inclusion criteria were: (a) peer-reviewed articles that developed and evaluated predictive ML models for valvular heart diseases; (b) published in English; (c) published in the last 10 years (covering 2014-2025); (d) studies that report relevant metric scores; (e) studies that developed models using adult patients (age 18 years and older). For disease type, we followed the valve-lesion matrix used by the American Heart Association/American College of Cardiology, which include aortic stenosis (AS), aortic regurgitation (AR), mitral stenosis (MS), mitral regurgitation (MR), tricuspid stenosis (TS), tricuspid regurgitation (TR), pulmonic stenosis (PS), and pulmonic regurgitation (PR)(1,2). Our exclusion criteria were: (a) non-original research (e.g., commentaries, editorials, reviews) or secondary research; (b) non-predictive ML models (e.g., segmentation, classification of physiological parameters, image enhancement). We also excluded studies conducted in animal models, preclinical settings, or in silico studies without application in human clinical data.

Supplementary Results

*Model Characteristics*

**Supplementary Figure 5** contains the distribution of identified best performing models in the included studies, as reported by the authors. CNN-based models were the most frequently identified models. Although tree-based methods are often grouped with classical machine learning, in this review we considered them separately because their non-parametric hierarchical structure and ensemble behaviour differ fundamentally from traditional parametric models. Of the studies analysed, 68% (n=133) developed and tested several model architectures, while 32% (n=62) reported developing and testing a single model architecture for each outcome. Of the included studies, a majority (79%; n=154) used only internal validation, while the others (21%; n=41) used both internal and external validation. Internal validation was done by either splitting the dataset or using resampling methods like k-fold cross validation, Monte Carlo cross validation, Leave-one-subject-out cross-validation, and bootstrapping. Some models developed using heart sounds from the 5-class dataset(3) were applied to a binary dataset(4) with label harmonization (for example, mapping all pathology classes to ‘abnormal’). We treated this as cross-dataset generalization analysis, not external validation. Many of the included studies used either proxy labels from publicly available databases (n=56; 28.7%) or expert adjudication (n=45; 23.1%) for outcome labelling. The full distribution and description of the sources of outcome labelling are shown in **Supplementary Figure 2** and **Supplementary Table 4** respectively.

Subsequently, 88.7% (n=173) of the studies reported multiple performance metrics for their models while the rest reported only a single metric. Of the included studies, 38.5% (n=75) used post-hoc explainability tools to interpret their model outcomes. Feature importance and Shapley Additive exPlanations (SHAP) were the most used methods. Tison et al.(5) employed a custom, parallel segment-based ML framework for post-hoc explainability, systematically quantifying the contribution of discrete ECG time-lead segments to the model’s predictions. The list of methods used are contained in Supplementary **Table 6**. Two studies(6,7) had both publicly available code and a dataset, while 70 had one or the other. 123 studies had neither. Of the studies analysed, 13% (n=26) compared model performance with existing predictive standards, such as the Euroscore II or the STS Mortality risk, or conducted a direct head-to-head evaluation against cardiologists using previously undiagnosed cases. In these 26 studies, the ML models showed superior performance, outperforming existing risk scores in 21 cases and cardiologists' diagnoses in 5 cases.


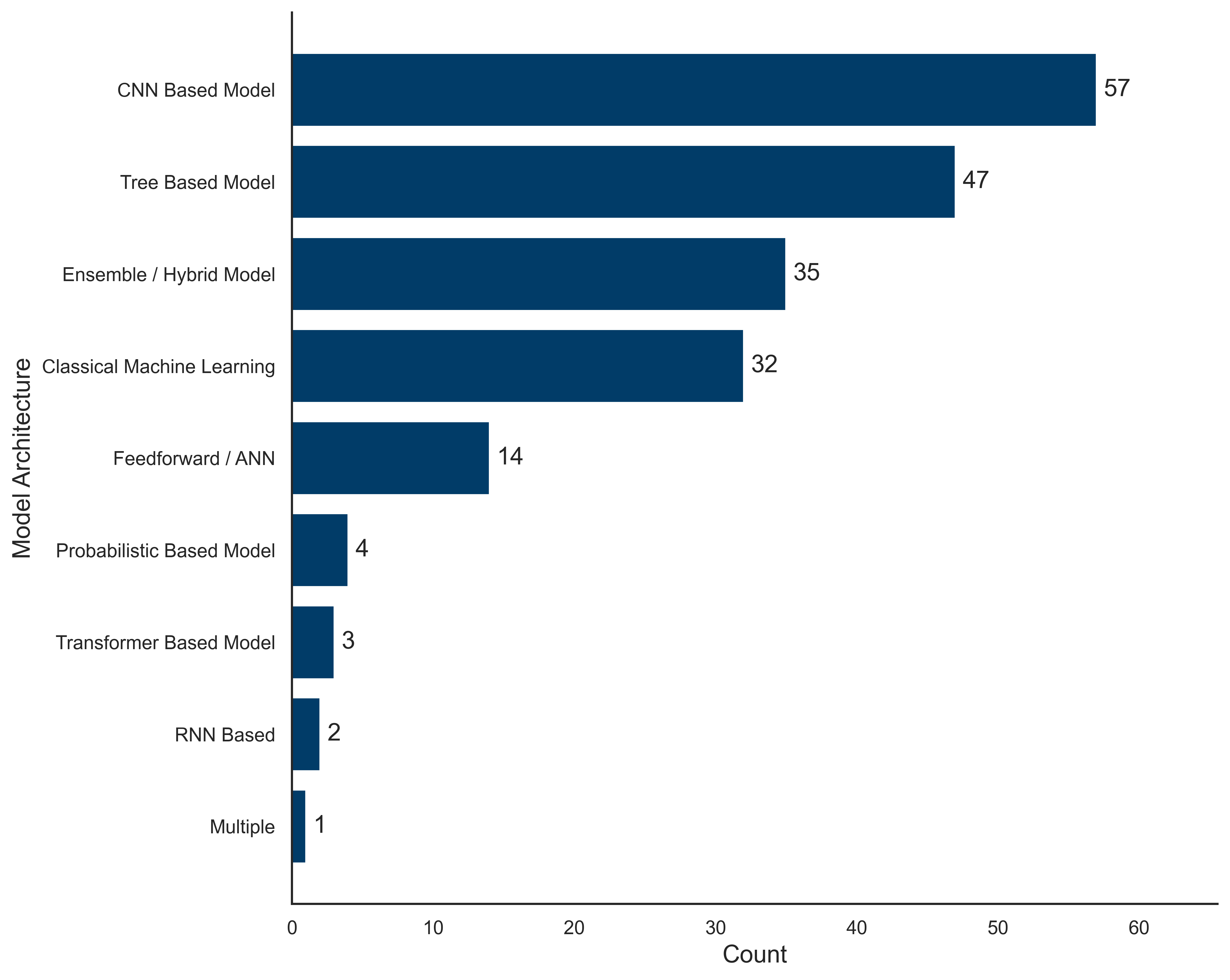


Supplementary Figure 5 | Model architecture of best-performing models.

Supplementary Table 6 | Post-hoc explainability methods of included studies

| Post-hoc method used | Number of studies |
| --- | --- |
| Feature importance | 24 |
| Shapley Additive exPlanations (SHAP) | 19 |
| Partial dependence plots | 2 |
| Gradient-weighted Class Activation Mapping (GRAD-CAM) | 16 |
| Saliency Maps | 5 |
| Backprop-based Feature Attribution (Captum, DeepLIFT) | 2 |
| Custom tools | 3 |
| Multiple | 4 |

**Supplementary References**

1. null n, Jneid H, Chikwe J et al. 2024 ACC/AHA Clinical Performance and Quality Measures for Adults With Valvular and Structural Heart Disease. JACC 2024;83:1579-1613.

2. null n, Otto Catherine M, Nishimura Rick A et al. 2020 ACC/AHA Guideline for the Management of Patients With Valvular Heart Disease. JACC 2021;77:e25-e197.

3. Yaseen, Son G-Y, Kwon S. Classification of Heart Sound Signal Using Multiple Features. Applied Sciences, 2018.

4. Liu C, Springer D, Li Q et al. An open access database for the evaluation of heart sound algorithms. Physiological Measurement 2016;37:2181.

5. Tison GH, Abreau S, Barrios J et al. Identifying Mitral Valve Prolapse at Risk for Arrhythmias and Fibrosis From Electrocardiograms Using Deep Learning. JACC Advances 2023;2.

6. Xiao K, Learned-Miller E, Kalogerakis E, Priest J, Fiterau M. Machine Learning for Automated Mitral Regurgitation Detection from Cardiac Imaging. MEDICAL IMAGE COMPUTING AND COMPUTER ASSISTED INTERVENTION, MICCAI 2023, PT VII. ["Univ Massachusetts Amherst, Amherst, MA 01003 USA", "Stanford Univ, Stanford, CA 94305 USA"], 2023:236-246.

7. Wang M, Guo B, Hu Y, Zhao Z, Liu C, Tang H. Transfer Learning Models for Detecting Six Categories of Phonocardiogram Recordings. J Cardiovasc Dev Dis 2022;9:86.
